# Supplementary material for: ‘ARVs are a constant reminder of lost freedom, whereas for others, they are liberating’: understanding the treatment narrative among people living with HIV (PLHIV) in Malawi and Zimbabwe–a qualitative study
Source: BMJ Open. 2024 Feb 28;14(2):e063138. doi: 10.1136/bmjopen-2022-063138 (PMC10910567; doi:10.1136/bmjopen-2022-063138)
Supplement: Supplementary data [file bmjopen-2022-063138supp001.pdf]

Supplementary Table 1

Country		Zimbabwe					Malawi					Sub Total
Region			Harare	Bulawayo	Midlands	Masvingo		Lilongwe	Blantyre	Thylo	Mangochi	
PLHIV	Age group	18-21	2	1	0	1		1	1	0	0	6
		22-25	0	0	1	0		0	2	1	0	4
		26-30	0	2	1	1		0	0	0	0	4
		31-35	1	0	1	1		2	3	1	3	12
		**Total**										**26**
HCPS	Gender	Female	2	3	2	1		2	2	3	2	17
		Male	1	0	1	2		1	3	3	1	12
	Job Title	Peer Counsellor	1	1	1	1	Expert Client	2	1	2	1	10
		Integrated Care Nurses		1		1	Clinical Officer	1	2	2	1	8
		ART Nurse	1	1	1	1	ART Nurse	2	0	1	1	
		Sexual Reproductive Health(SRH) Nurses	1		1		HIV Testing Services (HTS) Nurse	0	1	0	0	3
							HTS Focus Person	0	1	0	0	1
		**Total**										**29**
Influencers	Type of Role	Maternal Figure	1	1	1	1	Maternal Figure	1	1	1	1	8
		WhatsApp Group Leaders	1	1	1	1	WhatsApp Group Leaders	1	1	1	1	8
		Workshop Coordinators	1	1	1	1	Religious Leaders/Pastors	1	1	1	1	8
		Total										**24**
