# Supplementary material for: ‘ARVs are a constant reminder of lost freedom, whereas for others, they are liberating’: understanding the treatment narrative among people living with HIV (PLHIV) in Malawi and Zimbabwe–a qualitative study
Source: BMJ Open. 2024 Feb 28;14(2):e063138. doi: 10.1136/bmjopen-2022-063138 (PMC10910567; doi:10.1136/bmjopen-2022-063138)
Supplement: Author's manuscript [file bmjopen-2022-063138.draft_revisions.pdf]

BMJ Open

BMJ Open is committed to open peer review. As part of this commitment we make the peer review history of every article we publish publicly available.

When an article is published we post the peer reviewers' comments and the authors' responses online. We also post the versions of the paper that were used during peer review. These are the versions that the peer review comments apply to.

The versions of the paper that follow are the versions that were submitted during the peer review process. They are not the versions of record or the final published versions. They should not be cited or distributed as the published version of this manuscript.

BMJ Open is an open access journal and the full, final, typeset and author-corrected version of record of the manuscript is available on our site with no access controls, subscription charges or pay-per-view fees (http://bmjopen.bmj.com).

If you have any questions on BMJ Open's open peer review process please email info.bmjopen@bmj.com

BMJ Open

"ARVs are a constant reminder of lost freedom whereas for others they are liberating": Understanding the Treatment Narrative among People Living with HIV (PLHIV) in Malawi and Zimbabwe

Journal:	*BMJ Open*
Manuscript ID	bmjopen-2022-063138
Article Type:	Original research
Date Submitted by the Author:	26-Mar-2022
Complete List of Authors:	Gumede-Moyo, Sehlulekile; Ipsos MORI UK Ltd, Healthcare; London School of Hygiene & Tropical Medicine, Epidemiology and Population Health<br>Sharma, Sunny ; Ipsos MORI UK Ltd<br>Gwanzura, Clorata; Ministry of Health and Child Care<br>Nyirenda, Rose; Ministry of Health and Population Malawi<br>Mkandawire, Philip ; Population Services International<br>Chatora, Kumbirai ; Population Services International
Keywords:	PUBLIC HEALTH, QUALITATIVE RESEARCH, HIV & AIDS < INFECTIOUS DISEASES

SCHOLARONE™
Manuscripts

I, the Submitting Author has the right to grant and does grant on behalf of all authors of the Work (as defined in the below author licence), an exclusive licence and/or a non-exclusive licence for contributions from authors who are: i) UK Crown employees; ii) where BMJ has agreed a CC-BY licence shall apply, and/or iii) in accordance with the terms applicable for US Federal Government officers or employees acting as part of their official duties; on a worldwide, perpetual, irrevocable, royalty-free basis to BMJ Publishing Group Ltd ("BMJ") its licensees and where the relevant Journal is co-owned by BMJ to the co-owners of the Journal, to publish the Work in this journal and any other BMJ products and to exploit all rights, as set out in our licence.

The Submitting Author accepts and understands that any supply made under these terms is made by BMJ to the Submitting Author unless you are acting as an employee on behalf of your employer or a postgraduate student of an affiliated institution which is paying any applicable article publishing charge ("APC") for Open Access articles. Where the Submitting Author wishes to make the Work available on an Open Access basis (and intends to pay the relevant APC), the terms of reuse of such Open Access shall be governed by a Creative Commons licence – details of these licences and which Creative Commons licence will apply to this Work are set out in our licence referred to above.

Other than as permitted in any relevant BMJ Author's Self Archiving Policies, I confirm this Work has not been accepted for publication elsewhere, is not being considered for publication elsewhere and does not duplicate material already published. I confirm all authors consent to publication of this Work and authorise the granting of this licence.

For peer review only

‘*ARVs are a constant reminder of lost freedom whereas for others they are liberating*’: Understanding the Treatment Narrative among People Living with HIV (PLHIV) in Malawi and Zimbabwe

Sehlulekile Gumede-Moyo [1,2§]; Sunny Sharma[1]; Clorata Gwanzura[3]; Rose Nyirenda[4]; Philip Mkandawire[5] and Kumbirai Chatora [6]

1. Ipsos Healthcare, London, United Kingdom,

2. Department of Population Health, Faculty of Epidemiology and Population Health, London School of Hygiene and Tropical Medicine, London, UK

3. Department of AIDS & TB, Ministry of Health and Child Care, Harare, Zimbabwe

4. Department of HIV & AIDS, Ministry of Health and Population, Malawi

5. Population Services International, Malawi.

6. Population Services International, Zimbabwe.

[§]Corresponding author: Sehlulekile Gumede-Moyo, PhD, MPH, MBA. (seh.sokhela@gmail.com)

Key words: PLHIV; viral suppression; empathy; support; connection; campaigns.

For peer review only

Abstract

Objective: The research seek to deliver foundational insights on the current state of awareness of viral suppression and treatment as prevention, including the awareness and motivations of PLHIV, their influencers, and healthcare providers to stick with, counsel, or support taking their medicine every day. These insights will contribute to the messaging developed for a campaign across sub-Saharan Africa, but primarily Malawi and Zimbabwe.

Design: This was a qualitative study conducted in order to build an understanding of unknown parts of the HIV treatment journey through in-depth interviews.

Setting and Participants: Study participants were HIV-positive women, their influencers (e.g. parents/ partners/ siblings/ aunts and uncles/ religious leaders etc.), and healthcare providers from a range of regions in Zimbabwe and Malawi.

Results: A total of 26 young PLHIV, 29 HCPs, and 24 influencers such as maternal figures in the community were interviewed. Two main broad insights were drawn i.e., key needs and wider contextual factors. The key needs of young PLHIV to stay on treatment were empathy, support, and emotional connection with ART whilst the wider contextual factors impacting their treatment journeys were structural challenges and cultural reference points. Fear of shame and humiliation can also be significant barriers to disclosure and treatment. The fear pre-disposes the PLHIV to the need for empathy, support, and connection with ART. Mental health and anxiety appear to be co-morbid with HIV. Some PLHIV have a small and limited support network leading to very few people living openly with HIV. There is no "one size fits all" approach and maximizing campaign reach will likely need a multifaceted approach.

Conclusion: Currently the relationship between nurses and PLHIV can appear to be transactional. Through learning about the community (incl. PLHIV), we have more chances of communicating in a way that resonates.

For peer review only

For peer review only - http://bmjopen.bmj.com/site/about/guidelines.xhtml

What is already known on this topic?

- People living with HIV (PLHIV) cannot sexually transmit HIV to others when their viral load is suppressed.
- Combined approaches to scale up HIV prevention make a sustained impact on global HIV incidence rates.
- The existing HIV treatment campaigns in sub-Saharan Africa (SSA) are one-dimensional when it comes to the benefits of treatment.

What this study adds

- Knowledge on the current state of awareness of the benefits of treatment, and awareness of PLHIV's personal viral suppression status / viral load.
- Insights on the motivations for target groups to be virally suppressed and/or to take their medicine every day for life.

How this study might affect research, practice or policy

- Contribute to the messaging developed for a campaign across sub-Saharan Africa, but primarily Malawi and Zimbabwe
- To shift perspectives of PLHIV to take action based on the new concept / narrative of treatment literacy.

For peer review only

For peer review only - http://bmjopen.bmj.com/site/about/guidelines.xhtml

1. Introduction

The UNAIDS 95-95-95 targets aim to achieve a 95% level of diagnosis of people living with HIV (PLHIV) 95% of those diagnosed on antiretroviral therapy (ART), and 95% of those on ART virally suppressed by 2025 (1-4). In 2020, of all PLHIV globally, 84% knew their status, 73% (57–88%) were accessing treatment and 66% (53–79%) were virally suppressed (5). East and Southern Africa are the regions most affected by HIV are home to the largest numbers of PLHIV (6). While the number of PLHIV in East and Southern Africa continues to increase, access to ART is increasing as well (7). One goal of HIV care and treatment is achieving an undetectable viral load as it confers benefits at both the individual and public health-level (8, 9). There is strong evidence that supports that PLHIV cannot sexually transmit HIV to others when their viral load is suppressed to 200 HIV RNA copies/mL or lower, and they have been adherent to ART for at least 6 months (8, 10-12).

UNAIDS have called for combined approaches to scale up HIV prevention, to reinvigorate the global response and make a sustained impact on global HIV incidence rates (13). Combining separate strategies for different indications into singular prevention products can reduce the stigma around HIV and STI prevention, improve acceptability of and adherence to more convenient products, and be more cost-effective by addressing overlapping risks (14, 15). All combination prevention programmes require a strong community empowerment element and specific efforts to address legal and policy barriers, as well as the strengthening of health and social protection systems, plus actions to address gender inequality, stigma and discrimination (16, 17).

The majority of the existing HIV treatment campaigns in sub-Saharan Africa (SSA) are one-dimensional when it comes to the benefits of treatment, the fact that treatment extends life being the key benefit, rather than also focusing on other factors which could resonate more personally with individual PLHIV. This research sought to answer the following questions: 1), what is the current state of awareness of the benefits of treatment, 2) what is current state of awareness of PLHIV's personal viral suppression status / viral load, 3) What, if any, are the motivations for target groups to be virally suppressed and/or to take their medicine every day for life, 4) What is the current state of awareness of benefits of treatment and viral suppression, and 5) What are the opportunities for HCPs to understand and counsel on benefits of treatment + viral suppression?

For peer review only - http://bmjopen.bmj.com/site/about/guidelines.xhtml

2. Methods

2.1 Study Design

This was a qualitative study conducted in order to build an understanding of unknown parts of the HIV treatment journey through in-depth interviews with healthcare workers, young people living with HIV and their influencers (e.g. parents/ partners/ siblings/ aunts and uncles/ religious leaders etc.). The potential areas of interest were literacy and awareness of treatment; understanding their treatment journey (what is communicated to them about treatment, how well is it understood, what is compelling (if anything), barriers) and ideas around bringing a community approach to supporting PLHIV with treatment.

2.2 Study Setting

The research seek to deliver foundational insights on the current state of awareness of viral suppression and treatment as prevention, including the awareness and motivations of PLHIV, their influencers, and healthcare providers to stick with, counsel, or support taking their medicine every day. These insights will contribute to the messaging developed for a campaign across sub-Saharan Africa, but primarily Malawi and Zimbabwe that will aim to shift perspectives of PLHIV to take action based on the new concept / narrative of treatment literacy.

2.3 Sample recruitment

Our main study participants were HIV positive women, their influencers and healthcare providers from a range of regions in Zimbabwe and Malawi. The breakdown of study participants are indicated in Table 1. Eligibility criteria and recruitment procedures are described below.

2.3.1 PLHIV

The participants' names were retrieved from the Population Services International (PSI)-assisted ART clinic's database. The eligibility for the PLHIV was for one to be: 18-35 years old PLHIV (diagnosed at least 12 months ago); and not pregnant or breastfeeding. Quotas were set to recruit 1/3 of participants on treatment with good adherence, and 2/3 not taking treatment (non-adherence); a mix of respondents who had been on treatment for long and shorter periods of time, and a mix of those who were virally suppressed and those who were not.

2.3.2 HCPs

Eligibility criteria for HCP recruitment included having at least 3 years' experience in their current role, spending at least 50%.

For peer review only - http://bmjopen.bmj.com/site/about/guidelines.xhtml

Table 1: Study Participants

Country			Zimbabwe				Malawi					Sub Total
Region			Harare	Bulawayo	Midlands	Masvingo		Lilongwe	Blantyre	Thylo	Mangochi	
PLHIV	Age group	18-21	2	1	0	1		1	1	0	0	6
		22-25	0	0	1	0		0	2	1	0	4
		26-30	0	2	1	1		0	0	0	0	4
		31-35	1	0	1	1		2	3	1	3	12
		**Total**										**26**
HCPS	Gender	Female	2	3	2	1		2	2	3	2	17
		Male	1	0	1	2		1	3	3	1	12
	Job Title	Peer Counsellor	1	1	1	1	Expert Client	2	1	2	1	10
		Integrated Care Nurses		1		1	Clinical Officer	1	2	2	1	8
		ART Nurse	1	1	1	1	ART Nurse	2	0	1	1	
		Sexual Reproductive Health(SRH) Nurses	1		1		HIV Testing Services (HTS) Nurse	0	1	0	0	3
							HTS Focus Person	0	1	0	0	1
		**Total**										**29**
Influencers	Type of Role	Maternal Figure	1	1	1	1	Maternal Figure	1	1	1	1	8
		WhatsApp Group Leaders	1	1	1	1	WhatsApp Group Leaders	1	1	1	1	8
		Workshop Coordinators	1	1	1	1	Religious Leaders/Pastors	1	1	1	1	8
		Total										**24**

For peer review only

of their time working in HIV care, and interacting with at least 5 HIV+ women (aged 18-35) each day. HCPs worked at a variety of sites including public and private facilities.

2.3.3 Influencers

For this study an influencer was defined as someone who has an impact on PLHIV, either as part of their treatment journey or friends/relatives. The influencers were recruited after initial analysis of the PLHIV transcripts which determined who they were. These were maternal/grandmother figures, workshop leaders and WhatsApp group leaders. Workshops are support groups where PLHIV can meet to discuss concerns, ask questions, and receive peer support, workshop trainers are individuals that run these workshops. WhatsApp group chats are set up amongst communities to act as peer support networks consisting of PLHIV and their family/friends. WhatsApp group leaders are individuals that run these group chats in the communities. These influencers were recruited through snowballing.

2.4 Data collection

Data were collected from December 2020 to February 2021 in both countries. Semi-structured interviews were conducted by trained moderators with vast experience in qualitative data collection. Guides were produced in English, and then translated to Chichewa for Malawi, and into Ndebele and Shona for Zimbabwe. Study participants were asked before beginning the interview their language preference. Pretesting of the guides was done according to procedure. Due to the developing COVID-19 pandemic, planned face-to-face interviews were replaced by in-depth telephone interviews lasting approximately 45-75 minutes. If at any point phone lines were interrupted or participants had to pause/stop the interview, the interviewers arranged to call back at a suitable time. All interviews were audio-recorded for translation and transcription. Each participant was allocated with a unique personal identifier (ID) to maintain anonymity.

2.5 Data analysis

Smart transcript was used to group the responses in Excel. A hybrid (i.e., inductive and deductive) approach was used to conduct thematic analysis. Four research analysts (2 per country) independently coded the transcripts, after familiarising themselves with the scripts. The coded data were then shared with field managers who had field notes as additional sources

For peer review only - http://bmjopen.bmj.com/site/about/guidelines.xhtml

of data. In cases where there were discrepancies, all coders rechecked the transcripts and held meetings with the principal investigators and research managers until agreement was achieved. Anonymised quotes from some participants were also included in the manuscript to illustrate certain themes.

2.6 Patient and public involvement

The study participants were involved in the research recruitment, as they were the ones who led to the identification of influencers.

2.7 Ethical Clearance

Verbal explanation of the research purpose was given prior to conducting the interviews. All participants gave verbal consent over the phone for the recording and transcription of interviews which was documented on an informed consent form. The Ethical approval was obtained from the College of Medicine Research & Ethics Committee (COMREC) and Medical Research Council of Zimbabwe (MRCZ/A/2683).

For peer review only

3. Results

Two main broad insights were drawn from thematic analysis i.e., key needs and wider contextual factors. The key needs of young PLHIV are empathy, support and connection with ART whilst the wider contextual factors with impact in their treatment journeys are structural challenges and cultural hooks.

3.1 Key needs:

Fear of shame and humiliation can be significant barriers to disclosure and treatment. The fear pre-disposes the PLHIV to the need for empathy, support and connection with ART.

3.1.1. Empathy

There was disconnect between how HCPs and PLHIV view the perceptions of the disease in the community, which can act as a barrier to connection between messenger and patient. HCPs are an important messenger, however there can sometimes be a gap in understanding or lack of connection which can mean their messages are less credible to PLHIV.

"Life here is a bit difficult if people discover that you take medicine they either discriminate you or talk behind your back, so we don't disclose to people." - PLHIV, Malawi.

"Gone are the days when it was a taboo to talk about HIV but nowadays it's now common and people accept all the messages they hear about HIV however it differs from culture to

For peer review only - http://bmjopen.bmj.com/site/about/guidelines.xhtml

culture but generally people have accepted that HIV exists." – ART Nurse and midwife, Zimbabwe

Although HCPs say they treat PLHIV like 'anybody else' some PLHIV face discrimination in the community. For some, HIV status is not openly disclosed to the wider community as it can impact relationships.

"When it comes to professionals I guess they keep and maintain work ethics and professionalism, they treat us like any other person, they are supportive and encouraging compared to community at large." - PLHIV, Zimbabwe.

PLHIV often fear the worst and they often worry more about "what will people think?" rather than "what can I do to get better?" PLHIV expressed fear of judgement when telling a partner in case they were not accepting of their status, and not wanting to be seen taking medication and fear of rejection by the community.

Most of the PLHIV were more fearful of judgement as they do not want to be seen as promiscuous.

"People are so judgemental to the extent that they would say she got it through promiscuity" - PLHIV, Zimbabwe.

Initiating treatment at diagnosis is not always optimal because PLHIV are catastrophizing whilst nurses are providing a rational 'treatment seminar'. Test and Treat means that most patients are initiated to treatment straight away, but this is often not the best time to learn about treatment benefits and the importance of adherence as a patient's mental health (shock, numbness, fear, 'fight or flight', denial) makes it difficult to focus on information.

"It gave me a lot of stress, I didn't have peace and I was scared, should I tell my mom, should I start medication or I shouldn't start because of the rumours that the drugs deform people so I used to have a lot of doubts and it took me time to accept it, although at the hospital they said they weren't going to give me a chance to think about it, they said I had to start the very same day"- PLHIV, Malawi.

For peer review only - http://bmjopen.bmj.com/site/about/guidelines.xhtml

Taking treatment is a sign of HIV-positive status so therefore PLHIV need to hide their medication, however treatment can also be a reliever enabling the patient to show no signs of infection. PLHIV do not want members of the community knowing their status, so they lead a 'double life' to prevent discrimination.

"I feel like I am not different from others because I have no signs that shows that I am infected with the virus. At the same time this virus distanced me from my friends that I used to associate with. It changed my entire lifestyle because at the present moment I am living in a lie because I don't want people to know that I am infected." - PLHIV, Zimbabwe.

Religious influence plays a major role in people's lives and the perception of PLHIV within the community. Some religious communities are reluctant to talk about HIV which may contribute to preconceptions towards the disease and further prevent disclosure or adherence.. However, as HIV is not regularly spoken about in church, PLHIV fear that they will not receive the empathy they need to pull back from catastrophe in the church, nor does the church help overcome a major hurdle- spreading the message that all people including those with HIV are worthy of love equally under God.

"Many people believe that when one is HIV positive that person was very promiscuous, that is why people prefer to make it a secret about their status they do not want to be judged. Even in the community and some church organizations they do not want to talk about the virus. "- ART Nurse, Zimbabwe.

For some, ARVs are a constant reminder of lost freedom whereas for others it is liberating. *"I always think that if not for this disease, I could not have been taking this medicine and I could have been a free person."* – PLHIV, Malawi

"Living with HIV doesn't mean you are dying or you are disabled it's just a condition which you can live with, and doesn't affect your lifestyle as long you take treatment as prescribed."- PLHIV, Zimbabwe.

For peer review only - http://bmjopen.bmj.com/site/about/guidelines.xhtml

3.1.2. Support

Often PLHIV are in shock when they receive their diagnosis, and the patients need support to accept their status to begin treatment.

"When one gets tested they are given some shallow counselling which is superficial, and start treatment, the treatment becomes very difficult because the person will not have understood, and they are still in shock maybe because they were not expecting that result."- ART Nurse & Midwife, Zimbabwe

Helping people accept their status is a key challenge, the sooner people accept their status the more likely they are to adhere and move on with their life.

"I was worried because what I did not expect in my life has happened. But through meeting my good friends with good counselling things started changing"- PLHIV, Malawi.

Many are concerned about disclosing to their partner and family, but when they have a positive response (most do), this makes living with the disease much easier as they have support from people around them.

"I want to thank my young sister for support and encouragement she gave me, as young as she was, she was my pillar of strength even at a time when I wanted to quit, stood with me through out"- PLHIV, Zimbabwe.

Depression and anxiety can be a significant disruptor but there is little mental health support. Treating the PLHIV holistically beyond suppressing the HIV virus alone is important to maintain adherence.

"I was depressed because of my HIV status, it stressed me a lot. Even now when I think about it deeply it still stresses me"- PLHIV, Zimbabwe.

For peer review only

For peer review only - http://bmjopen.bmj.com/site/about/guidelines.xhtml

3.1.3 Connection with ART

HCPs felt that there was good treatment literacy in both Zimbabwe and Malawi. They demonstrated significant HIV knowledge and were confident communicating messages to patients. Gaps noted by HCPs were that some PLHIV struggling with adherence tended to do so because they did not understand disease severity or benefits of medication. Knowledge of viral suppression varies across countries, but it is a motivating message. Although, there is a good basic level of knowledge around treatment benefits across all PLHIV.

HCPs in Zimbabwe did not widely communicate information about viral suppression. PLHIV clearly communicated the benefits of treatment and what motivated them to adhere (being healthy, controlling the virus, living a normal life, not looking like they have HIV to the community) but there were very few mentions of viral suppression or reduction in viral load.

In Malawi, some HCPs communicated information about viral suppression to PLHIV and PLHIV had a reasonable understanding of the concept. They talked about the fact that you could reach a point where the virus is not evident in your bloodstream and know that this means you will not transfer the virus to your sexual partner or child.

HCPs saw the effectiveness and impact that understanding viral suppression could have on driving behaviour-change and adherence to treatment. Especially when PLHIV are able to link viral suppression to feeling better, they become more motivated to adhere to treatment.

"They get motivated…if we tell a client that your levels are undetectable and if you continue taking medication like that you will have a long time before getting sick. Once a client hears that, they get motivated and make promises that they will maintain taking medication properly." – Clinical officer, Malawi.

3.2 Contextual Factors.

3.2.1 Structural Challenges

Poverty-related challenges remain a central barrier to adherence and the COVID-19 pandemic made the situation worse (Figure 1). PLHIV cannot take their medication if they do not have enough food to eat, even if they are motivated to do so. Sourcing food becomes core

For peer review only

For peer review only - http://bmjopen.bmj.com/site/about/guidelines.xhtml

priority and HIV care is down-prioritized. COVID-19 has exacerbated existing financial challenges.

"These people must be a priority because it can be anyone, you mother, brother sister or friend. These people need care so that they do not affect others. During budget allocation the government must prioritise the Health sector and avail funds towards their welfare and resources as first preferences. I believe everyone has a role to play in the care of PLHIV."- Influencer, (maternal figure), Zimbabwe

Some PLHIVs were not able to afford transport to the clinics leading to missed appointments, which affected treatment schedule. This was a particular challenge in rural areas and mentioned more in Malawi.

"It is difficult when patients miss appointments and don't take their medication as their viral load gets higher and you have to spend more time educating them about taking treatment properly." – Client Officer, Malawi

There is also lack of resources in health facilities. Most health facilities do not have enough staff, meaning that less time is spent educating patients. Resource challenges include electricity blackouts, inconsistent condom supply, and occasional low stock of medications.

"It's a challenge because sometimes the ART may not be available, sometimes even the testing kits are not available, and samples are failing to go to the lab on time, end up getting stale due to lack of resources. Initially, we are supposed to retest and do Cd4 counts tests every now and then to know the patient's viral load, but unfortunately we are failing to do that. We watch people deteriorate because we have nothing to help them with."- ART Nurse, Zimbabwe

I could have recommended that they give us more nurses so that people get helped faster."- ART Nurse, Malawi

PLHIV stated that overly technical language - information from HCP may not be understood, unless explained in terms which are relatable. Some PLHIV struggle to read information leaflets, posters, and other written communication about HIV because they are too technical.

For peer review only - http://bmjopen.bmj.com/site/about/guidelines.xhtml

3.2.2 Cultural Hooks

People in Malawi and Zimbabwe are diverse with different hobbies and interests. There is no "one size fits all" approach and maximising campaign reach will likely need a multifaceted approach.

Young PLHIV may struggle with their disease but still find ways to have fun and live life. They have identified female role models who represent independence, open-mindedness, and progress. There is a need to show PLHIV that they too can be educated, independent, and valued members of society – just like their role models. These role models embody PLHIV's aspirations and dreams, which are focused on "thriving" – becoming independent, providers, and being respected by their families and communities.

I think this must be done by everyone because people trust different people, it should be an issue discussed at household level, community, all HCPs, NGOs and social media – influencer (trainer) – Zimbabwe

Some people think HIV ended and are becoming careless. So it's important that we are not quiet, we have to do awareness campaigns, encouraging people, reminding them about HIV and its dangers. – Influencer (Religious leader) Malawi.

5. Discussion

This study provided some foundational insights into the current state of awareness of viral suppression and treatment as prevention, including the awareness and motivations of PLHIV, their influencers, and healthcare providers. The key themes demonstrated that there is a need for empathy, and for attention to be paid to wider contextual factors in communications campaigns.

Early life experiences of HIV are scary and/or traumatic. Associations include loved ones being 'taken away' and not returning, seeing people in the community who are sick & frail whilst people say they have AIDS as a catch all for any serious illness (18, 19). For healthcare workers specifically ART nurses, treating HIV might feel like a routine but for every individual PLHIV, it represents a profound change in their lives often characterised by fear, shock, sorrow, loss and low self-worth (20, 21). Our study revealed a disconnect between how HCPs and PLHIV view the perceptions of the disease in the community which

For peer review only - http://bmjopen.bmj.com/site/about/guidelines.xhtml

can act as a barrier towards connection between messenger and patient. Focusing too heavily on educating and getting PLHIV onto treatment can mean that HCPs fail to meet the emotional needs of PLHIV, leaving them feeling even more overwhelmed and deflated.

There is a need to create an opportunity for the HCP-PLHIV interaction to become more transformational, rather than transactional. HCPs tend to be focused on educating to make sure PLHIV are well informed on HIV; encouraging disclosure; tracing contacts and adherence to treatment. HCPs see their role primarily as educators, secondary comes listening. On the other hand PLHIV have a lot to process in terms of diagnosis; what implications this has for their life and those around them and the new information on HIV and care.

HCPs should be orientated on the fact that rationalities regarding treatment-taking extend beyond the biomedical realm, requiring adjustments to sense of self and identity, and decision-making that is situated and socially embedded (21-23). However, in many clinical centres, standards and guidelines surrounding HIV treatment communication may be absent or lack specificity, which is a hindrance (23). HCPs can be trained to understand the types of support different patients need at different times. Treatment literacy campaigns should also consider how to still resonate with people when they are going through tough times.

It is important to understand that some PLHIV 'catastrophize' every potential disclosure of their status, particularly when out of their control. In times of hardship, people in Malawi and Zimbabwe turn to the church as a message of hope and comfort for a better future. HIV is a traumatic experience for PLHIV and they can feel like they have committed a sin. As HIV is not spoken about at a community level, they are scared that they will be shunned by all. HCPs are already using a range of communication tools to convey viral suppression and treatment benefits in an accessible way. This was mirrored by PLHIV when expressing their knowledge of HIV. These tools could be used to scale-up the viral suppression message and reach a point where it's well recognized and understood by not just PLHIV, but the wider community too. Studies conducted in South Africa, Malawi and Kenya among health providers reported mixed attitudes about treatment as prevention although most were supportive (24-27). In Malawi, HIV care providers and program stakeholders expressed concerns related to: equating 'undetectable' with 'healed', which may impact adherence negatively, and to a potential increase in promiscuity and HIV re-infection (25). Kenyan

For peer review only - http://bmjopen.bmj.com/site/about/guidelines.xhtml

health providers reported fears that telling patients undetectable viral loads lead to other risk behaviours, or that consequent HIV transmission would be blamed on them (26).

Busy clinics can mean less time spent with PLHIV and can result in gaps of education. Patients who are defaulting tend to do so because they do not understand disease severity or benefits of medication. PLHIV may still be in denial stage at the time of diagnosis and treatment. Patients are not in the right frame of mind to absorb key messages about benefits and the importance of adherence at initial treatment stage. Our findings confirm the health systems barriers such as limited and unprofessional providers at healthcare facilities, un-availability of drugs, and long travel distance that have been identified by other researchers (23, 28-31).

Strengths and Limitations of the study

We obtained a rich and nuanced appreciation of the current state of awareness of viral suppression and treatment as prevention, including the awareness and motivations as we sampled from a range of PLHIV, their influencers, and healthcare providers. The depth of inquiry allowed us to identify potential areas of interest were literacy and awareness of treatment; treatment journey and ideas around bringing a community approach to supporting PLHIV with treatment. These issues were also considered in depth through a unique lens of influencers which were sampled from a variety of positions within the communities to allow a broad review.

Caution should be exercised when interpreting the results of this exploratory analysis due to small sample size and limited breadth of influencers sampled. Several campaigns have reinforced how people 'should' feel about PLHIV hence HCPs and influencers may have answered questions based on what they think is 'correct' or 'acceptable', rather than their true thoughts or beliefs which may be undesirable. The insights of the study is likely to contribute to the development of campaigns across sub-Saharan Africa, and creating of messaging that can go beyond the medical and PLHIV living a longer life.

6. Conclusion

Treatment literacy campaigns should also consider how to still resonate with people when they are going through tough times. Currently, the relationship between nurses and PLHIV can appear to be transactional. Some HCPs are hesitant to speak about viral suppression denoting

For peer review only - http://bmjopen.bmj.com/site/about/guidelines.xhtml

the end of onward transmission risk. There is a need to show PLHIV that they too can be independent, and valued members of society. Through learning about the community (incl. PLHIV), we have more chance of communicating in a way that resonates.

Authors' contributions

The manuscript was drafted by SGM and SS with contributions from all the authors. All authors read and approved the final manuscript.

Availability of Data

This study used data obtained from human participants. The dataset (anonymized survey responses) is owned by the governments of Malawi and Zimbabwe.

Competing interests

None declared.

Acknowledgements

This work was supported with leadership by the Ministry of Health and Population in Malawi and in Ministry of Health and Child Care (MoHCC) in Zimbabwe. We thank our partners Population Services International in Malawi and Zimbabwe for collaborating on the implementation of this research.

Funding Statement

This study was supported by the Flip the Script Project funded by the Johnson & Johnson and Bill & Melinda Gates Foundation. The funders were involved in the study design, but had no role in data collection and interpretation, or the decision to submit the work for publication.

For peer review only - http://bmjopen.bmj.com/site/about/guidelines.xhtml

References

1. UNAIDS. 90-90-90: An ambitious target to help end the AIDS epidemic. Geneva; 2014.
2. UNAIDS. Political Declaration on HIV and AIDS: On the Fast Track to Accelerating the Fight against HIV and to Ending the AIDS Epidemic by 2030. Geneva; 2016.
3. UNAIDS. Understanding Fast Track: Accelerating Action to end the AIDS pandemic by 2030 2015.
4. UNAIDS. 2025 AIDS TARGETS Second face-to-face meeting of the Steering Committee on Target Setting, Impact, and Resource Needs. 2019.
5. UNAIDS. UNAIDS Fact Sheet:Preliminary UNAIDS 2021 epidemiological estimates. New York, USA. ; 2021.
6. UNAIDS Data: East and Southern Africa 90-90-90 progress [Internet]. 2021. Available from: https://esahub.unaids.org/.
7. AIDSinfo. Global data on HIV epidemiology and response. 2021.
8. Diepstra K, Lu H, McManus KA, Rogawski McQuade ET, Rhodes AG, Westreich D. What we talk about when we talk about durable viral suppression. AIDS (London, England). 2020;34(11):1683-6.
9. Eisinger RW, Dieffenbach CW, Fauci AS. HIV Viral Load and Transmissibility of HIV Infection: Undetectable Equals Untransmittable. Jama. 2019;321(5):451-2.
10. U=U taking off in 2017 [press release]. LANCET2017.
11. U=U Consensus Statement [press release]. 2021.
12. Bhatt SJ, Douglas N. Undetectable equals untransmittable (U = U): implications for preconception counseling for human immunodeficiency virus serodiscordant couples. American journal of obstetrics and gynecology. 2020;222(1):53.e1-.e4.
13. UNAIDS. Combination HIV Prevention: Tailoring and Coordinating Biomedical, Behavioural and Structural Strategies to Reduce New HIV Infections. Geneva2010.
14. Young Holt B, Dellplain L, Creinin MD, Peine KJ, Romano J, Hemmerling A. A strategic action framework for multipurpose prevention technologies combining contraceptive hormones and antiretroviral drugs to prevent pregnancy and HIV. Eur J Contracept Reprod Health Care. 2018;23(5):326-34.
15. Schaefer R, Gregson S, Fearon E, Hensen B, Hallett TB, Hargreaves JR. HIV prevention cascades: a unifying framework to replicate the successes of treatment cascades. The Lancet HIV. 2019;6(1):e60-e6.
16. UNAIDS. Prevention Gap Report. 2016.
17. Baral S, Rao A, Sullivan P, Phaswana-Mafuya N, Diouf D, Millett G, et al. The disconnect between individual-level and population-level HIV prevention benefits of antiretroviral treatment. Lancet HIV. 2019;6(9):e632-e8.
18. Keuroghlian AS, Kamen CS, Neri E, Lee S, Liu R, Gore-Felton C. Trauma, dissociation, and antiretroviral adherence among persons living with HIV/AIDS. J Psychiatr Res. 2011;45(7):942-8.
19. Seedat S. Interventions to Improve Psychological Functioning and Health Outcomes of HIV-Infected Individuals with a History of Trauma or PTSD. Current HIV/AIDS Reports. 2012;9(4):344-50.
20. Horter S, Thabede Z, Dlamini V, Bernays S, Stringer B, Mazibuko S, et al. "Life is so easy on ART, once you accept it": Acceptance, denial and linkage to HIV care in Shiselweni, Swaziland. Social science & medicine (1982). 2017;176:52-9.
21. Horter S, Seeley J, Bernays S, Kerschberger B, Lukhele N, Wringe A. Dissonance of Choice: Biomedical and Lived Perspectives on HIV Treatment-Taking. Med Anthropol. 2020;39(8):675-88.

For peer review only - http://bmjopen.bmj.com/site/about/guidelines.xhtml

22. Calabrese SK, Mayer KH, Marcus JL. Prioritising pleasure and correcting misinformation in the era of U=U. Lancet HIV. 2021;8(3):e175-e80.
23. Calabrese SK, Mayer KH. Stigma impedes HIV prevention by stifling patient-provider communication about U = U. Journal of the International AIDS Society. 2020;23(7):e25559.
24. Zhou A. The uncertainty of treatment: Women's use of HIV treatment as prevention in Malawi. Social science & medicine (1982). 2016;158:52-60.
25. Berner-Rodoreda A, Dambe R, Ngwira E, Neuhann F, Bärnighausen T, S P. U=U - Catalyst or barrier for men‘s engagement in ART? Stakeholder views from Blantyre, Malawi. AIDS 20202020.
26. Ngure K, Ongolly F, Dolla A, Awour M, Mugwanya KK, Irungu E, et al. "I just believe there is a risk" understanding of undetectable equals untransmissible (U = U) among health providers and HIV-negative partners in serodiscordant relationships in Kenya. Journal of the International AIDS Society. 2020;23(3):e25466.
27. Lippman SA, West R, Gómez-Olivé FX, Leslie HH, Twine R, Gottert A, et al. Treatment as Prevention-Provider Knowledge and Counseling Lag Behind Global Campaigns. Journal of acquired immune deficiency syndromes (1999). 2020;83(2):e9-e12.
28. Naanyu V, Ruff J, Goodrich S, Spira T, Bateganya M, Toroitich-Ruto C, et al. Qualitative exploration of perceived benefits of care and barriers influencing HIV care in trans Nzoia, Kenya. BMC health services research. 2020;20(1):355.
29. Croome N, Ahluwalia M, Hughes LD, Abas M. Patient-reported barriers and facilitators to antiretroviral adherence in sub-Saharan Africa. AIDS (London, England). 2017;31(7):995-1007.
30. Kagee AR, R.H.; Berkman, A.; Hoffman, S.; Campos, L.; Swartz, L. . Structural barriers to ART adherence in Southern Africa: Challenges and potential ways forward. . Global public health. 2011;6:83-97.
31. Lockman S, Holme MP, Makhema J, Bachanas P, Moore J, Wirth KE, et al. Implementation of Universal HIV Testing and Treatment to Reduce HIV Incidence in Botswana: the Ya Tsie Study. Curr HIV/AIDS Rep. 2020;17(5):478-86.

For peer review only - http://bmjopen.bmj.com/site/about/guidelines.xhtml

Figure 1:

Legend – Structural challenges to ART adherence

For peer review only

For peer review only - http://bmjopen.bmj.com/site/about/guidelines.xhtml

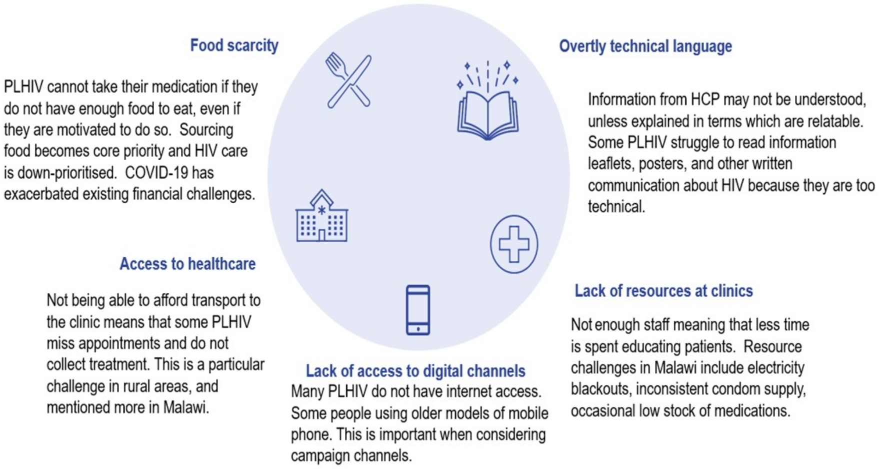

Figure 1: Structural challenges to ART adherence

Structural challenges to ART adherence

338x190mm (96 x 96 DPI)

BMJ Open

"ARVs are a constant reminder of lost freedom whereas for others they are liberating": Understanding the Treatment Narrative among People Living with HIV (PLHIV) in Malawi and Zimbabwe - A qualitative study

Journal:	*BMJ Open*
Manuscript ID	bmjopen-2022-063138.R1
Article Type:	Original research
Date Submitted by the Author:	23-Dec-2022
Complete List of Authors:	Gumede-Moyo, Sehlulekile; Ipsos MORI UK Ltd, Healthcare; London School of Hygiene & Tropical Medicine, Epidemiology and Population Health<br>Sharma, Sunny ; Ipsos MORI UK Ltd<br>Gwanzura, Clorata; Ministry of Health and Child Care<br>Nyirenda, Rose; Ministry of Health and Population Malawi<br>Mkandawire, Philip ; Population Services International<br>Chatora, Kumbirai ; Population Services International<br>Hasen, Nina; Population Services International, HIV and TB
**Primary Subject Heading**:	Evidence based practice
Secondary Subject Heading:	Epidemiology, Global health, Health services research, HIV/AIDS, Qualitative research
Keywords:	PUBLIC HEALTH, QUALITATIVE RESEARCH, HIV & AIDS < INFECTIOUS DISEASES

SCHOLARONE™
Manuscripts

I, the Submitting Author has the right to grant and does grant on behalf of all authors of the Work (as defined in the below author licence), an exclusive licence and/or a non-exclusive licence for contributions from authors who are: i) UK Crown employees; ii) where BMJ has agreed a CC-BY licence shall apply, and/or iii) in accordance with the terms applicable for US Federal Government officers or employees acting as part of their official duties; on a worldwide, perpetual, irrevocable, royalty-free basis to BMJ Publishing Group Ltd ("BMJ") its licensees and where the relevant Journal is co-owned by BMJ to the co-owners of the Journal, to publish the Work in this journal and any other BMJ products and to exploit all rights, as set out in our licence.

The Submitting Author accepts and understands that any supply made under these terms is made by BMJ to the Submitting Author unless you are acting as an employee on behalf of your employer or a postgraduate student of an affiliated institution which is paying any applicable article publishing charge ("APC") for Open Access articles. Where the Submitting Author wishes to make the Work available on an Open Access basis (and intends to pay the relevant APC), the terms of reuse of such Open Access shall be governed by a Creative Commons licence – details of these licences and which Creative Commons licence will apply to this Work are set out in our licence referred to above.

Other than as permitted in any relevant BMJ Author's Self Archiving Policies, I confirm this Work has not been accepted for publication elsewhere, is not being considered for publication elsewhere and does not duplicate material already published. I confirm all authors consent to publication of this Work and authorise the granting of this licence.

For peer review only

‘*ARVs are a constant reminder of lost freedom whereas for others they are liberating*’: Understanding the Treatment Narrative among People Living with HIV (PLHIV) in Malawi and Zimbabwe. A qualitative study.

Sehlulekile Gumede-Moyo [1,2§]; Sunny Sharma[1]; Clorata Gwanzura[3]; Rose Nyirenda[4]; Philip Mkandawire[5]; Kumbirai Chatora [6]and Nina Hasen[7]

1. Ipsos Healthcare, London, United Kingdom,

2. Department of Population Health, Faculty of Epidemiology and Population Health, London School of Hygiene and Tropical Medicine, London, UK

3. Department of AIDS & TB, Ministry of Health and Child Care, Harare, Zimbabwe

4. Department of HIV & AIDS, Ministry of Health and Population, Malawi

5. Population Services International, Malawi.

6. Population Services International, Zimbabwe.

7. Population Service International Global.

[§]Corresponding author: Sehlulekile Gumede-Moyo, PhD, MPH, MBA. (seh.sokhela@gmail.com)

Key words: PLHIV; viral suppression; empathy; support; connection; campaigns.

For peer review only - http://bmjopen.bmj.com/site/about/guidelines.xhtml

Abstract

Objective: The aim of the research was to understand what drives and motivates young women living with HIV in their treatment journeys, as well as their key influencers. The findings will assist in appreciating their obstacles to treatment and constructing the most effective ways to convey fresh messages to them. These insights will contribute to the messaging developed for a campaign across sub-Saharan Africa, but primarily Malawi and Zimbabwe.

Design: This was a qualitative study conducted in order to build an understanding of unknown parts of the HIV treatment journey through in-depth interviews. A hybrid approach was used to conduct thematic analysis.

Setting and Participants: Study participants were HIV-positive women, their influencers (e.g. parents/ partners/ siblings/ aunts and uncles/ religious leaders etc.), and healthcare providers from a range of regions in Zimbabwe and Malawi.

Results: A total of 26 young PLHIV, 29 HCPs, and 24 influencers such as maternal figures in the community were interviewed. Two main broad insights were drawn i.e., key needs and wider contextual factors. The key needs of young PLHIV to stay on treatment were empathy, support, and emotional connection with ART whilst the wider contextual factors impacting their treatment journeys were structural challenges and cultural reference points. Fear of shame and humiliation can also be significant barriers to disclosure and treatment. The fear pre-disposes the PLHIV to the need for empathy, support, and connection with ART. Mental health and anxiety appear to be co-morbid with HIV. Some PLHIV have a small and limited support network leading to very few people living openly with HIV. There is no "one size fits all" approach and maximizing campaign reach will likely need a multifaceted approach.

Conclusion: Currently the relationship between nurses and PLHIV can appear to be transactional. Through learning about the community (incl. PLHIV), there are more chances of communicating in a way that resonates.

For peer review only - http://bmjopen.bmj.com/site/about/guidelines.xhtml

Strengths

- The participants of the study were sampled from a range of PLHIV, their influencers, and healthcare providers.
- The depth of inquiry allowed to identify potential areas of interest were literacy and awareness of treatment.

Limitations

- Small sample size and limited breadth of influencers sampled

For peer review only

For peer review only - http://bmjopen.bmj.com/site/about/guidelines.xhtml

1. Introduction

The UNAIDS 95-95-95 targets aim to achieve a 95% level of diagnosis of people living with HIV (PLHIV) 95% of those diagnosed on antiretroviral therapy (ART), and 95% of those on ART virally suppressed by 2025 (1-4). In 2020, of all PLHIV globally, 84% knew their status, 73% (57–88%) were accessing treatment and 66% (53–79%) were virally suppressed (5). East and Southern Africa are the regions most affected by HIV are home to the largest numbers of PLHIV (6). While the number of PLHIV in East and Southern Africa continues to increase, access to ART is increasing as well (7). Young people, especially women and key populations, continue to have a disproportionately low level of viral suppression (4). One goal of HIV care and treatment is achieving an undetectable viral load as it confers benefits at both the individual and public health-level (8, 9). There is strong evidence that supports that PLHIV cannot sexually transmit HIV to others when their viral load is suppressed to 200 HIV RNA copies/mL or lower, and they have been adherent to ART for at least 6 months (8, 10-12).

UNAIDS have called for combined approaches to scale up HIV prevention, to reinvigorate the global response and make a sustained impact on global HIV incidence rates (13). Combining separate strategies for different indications into singular prevention products can reduce the stigma around HIV and STI prevention, improve acceptability of and adherence to more convenient products, and be more cost-effective by addressing overlapping risks (14, 15). All combination prevention programmes require a strong community empowerment element and specific efforts to address legal and policy barriers, as well as the strengthening of health and social protection systems, plus actions to address gender inequality, stigma and discrimination (16, 17).

The majority of the existing HIV treatment campaigns in sub-Saharan Africa (SSA) are one-dimensional when it comes to the benefits of treatment, the fact that treatment extends life being the key benefit, rather than also focusing on other factors which could resonate more personally with individual PLHIV. The aim of the research was to understand what drives and motivates young women living with HIV, as well as their key influencers. The findings will assist in appreciating their obstacles to treatment and constructing the most effective ways to convey fresh messages to them.

For peer review only - http://bmjopen.bmj.com/site/about/guidelines.xhtml

2. Methods

2.1 Study Design

This was a qualitative study conducted in order to build an understanding of unknown parts of the HIV treatment journey through in-depth interviews with healthcare workers, young people living with HIV and their influencers (e.g. parents/ partners/ siblings/ aunts and uncles/ religious leaders etc.). The potential areas of interest were literacy and awareness of treatment; understanding their treatment journey (what is communicated to them about treatment, how well is it understood, what is compelling (if anything), barriers) and ideas around bringing a community approach to supporting PLHIV with treatment.

2.2 Study Setting

The research seek to deliver foundational insights on the current state of awareness of viral suppression and treatment as prevention, including the awareness and motivations of PLHIV, their influencers, and healthcare providers to stick with, counsel, or support taking their medicine every day. These insights will contribute to the messaging developed for a campaign across sub-Saharan Africa, but primarily Malawi and Zimbabwe that will aim to shift perspectives of PLHIV to take action based on the new concept / narrative of treatment literacy.

2.3 Sample recruitment

Our main study participants were HIV positive women, their influencers and healthcare providers from a range of regions in Zimbabwe and Malawi. The breakdown of study participants are indicated in Table 1. Eligibility criteria and recruitment procedures are described below. Young HIV positive women (18-35 years) were targeted as they have disproportionately low levels of viral suppression based on UNAIDS reports (4-6) .

2.3.1 PLHIV

The participants' names were retrieved from the Population Services International (PSI)-assisted ART clinic's database. The eligibility for the PLHIV was for one to be: 18-35 years old PLHIV (diagnosed at least 12 months ago); and not pregnant or breastfeeding. Quotas were set to recruit 1/3 of participants on treatment with good adherence, and 2/3 not taking treatment (non-adherence); a mix of respondents who had been on treatment for long and shorter periods of time, and a mix of those who were virally suppressed and those who were not.

For peer review only - http://bmjopen.bmj.com/site/about/guidelines.xhtml

2.3.2 HCPs

Eligibility criteria for HCP recruitment included having at least 3 years' experience in their current role, spending at least 50% of their time working in HIV care, and interacting with at least 5 HIV+ women (aged 18-35) each day. HCPs worked at a variety of sites including public and private facilities.

2.3.3 Influencers

For this study an influencer was defined as someone who has an impact on PLHIV, either as part of their treatment journey or friends/relatives. The influencers were recruited after initial analysis of the PLHIV transcripts which determined who they were. These were maternal/grandmother figures, workshop leaders and WhatsApp group leaders. Workshops are support groups where PLHIV can meet to discuss concerns, ask questions, and receive peer support, workshop trainers are individuals that run these workshops. WhatsApp group chats are set up amongst communities to act as peer support networks consisting of PLHIV and their family/friends. WhatsApp group leaders are individuals that run these group chats in the communities. These influencers were recruited through snowballing.

2.4 Data collection

Data were collected from December 2020 to February 2021 in both countries. Semi-structured interviews were conducted by trained moderators with vast experience in qualitative data collection. Guides were produced in English, and then translated to Chichewa for Malawi, and into Ndebele and Shona for Zimbabwe. Study participants were asked before beginning the interview their language preference. Pretesting of the guides was done according to procedure. Due to the developing COVID-19 pandemic, planned face-to-face interviews were replaced by in-depth telephone interviews lasting approximately 45-75 minutes. If at any point phone lines were interrupted or participants had to pause/stop the interview, the interviewers arranged to call back at a suitable time. All interviews were audio-recorded for translation and transcription. Each participant was allocated with a unique personal identifier (ID) to maintain anonymity.

review only

For peer review only - http://bmjopen.bmj.com/site/about/guidelines.xhtml

Table 1: Study Participants

Country			Zimbabwe				Malawi					Sub Total
**Region**			**Harare**	**Bulawayo**	**Midlands**	**Masvingo**		**Lilongwe**	**Blantyre**	**Thylo**	**Mangochi**	
**PLHIV**	**Age group**	18-21	2	1	0	1		1	1	0	0	6
		22-25	0	0	1	0		0	2	1	0	4
		26-30	0	2	1	1		0	0	0	0	4
		31-35	1	0	1	1		2	3	1	3	12
		**Total**										**26**
**HCPS**	**Gender**	Female	2	3	2	1		2	2	3	2	17
		Male	1	0	1	2		1	3	3	1	12
	**Job Title**	Peer Counsellor	1	1	1	1	Expert Client	2	1	2	1	10
		Integrated Care Nurses		1		1	Clinical Officer	1	2	2	1	8
		ART Nurse	1	1	1	1	ART Nurse	2	0	1	1	
		Sexual Reproductive Health(SRH) Nurses	1		1		HIV Testing Services (HTS) Nurse	0	1	0	0	3
							HTS Focus Person	0	1	0	0	1
		**Total**										**29**
**Influencers**	**Type of Role**	Maternal Figure	1	1	1	1	Maternal Figure	1	1	1	1	8
		WhatsApp Group Leaders	1	1	1	1	WhatsApp Group Leaders	1	1	1	1	8
		Workshop Coordinators	1	1	1	1	Religious Leaders/Pastors	1	1	1	1	8
		Total										**24**

For peer review only - http://bmjopen.bmj.com/site/about/guidelines.xhtml

2.4 Data collection

Data were collected from December 2020 to February 2021 in both countries. Semi-structured interviews were conducted by trained moderators with vast experience in qualitative data collection. Guides were produced in English, and then translated to Chichewa for Malawi, and into Ndebele and Shona for Zimbabwe. Study participants were asked before beginning the interview their language preference. Pretesting of the guides was done according to procedure. Due to the developing COVID-19 pandemic, planned face-to-face interviews were replaced by in-depth telephone interviews lasting approximately 45-75 minutes. If at any point phone lines were interrupted or participants had to pause/stop the interview, the interviewers arranged to call back at a suitable time. All interviews were audio-recorded for translation and transcription. Each participant was allocated with a unique personal identifier (ID) to maintain anonymity.

2.5 Data analysis

Smart transcript was used to group the responses in Excel. A hybrid (i.e., inductive and deductive) approach was used to conduct thematic analysis. Four research analysts (2 per country) independently coded the transcripts, after familiarising themselves with the scripts. The coded data were then shared with field managers who had field notes as additional sources of data. In cases where there were discrepancies, all coders rechecked the transcripts and held meetings with the principal investigators and research managers until agreement was achieved. Anonymised quotes from some participants were also included in the manuscript to illustrate certain themes.

2.6 Patient and public involvement

The study participants were involved in the research recruitment, as they were the ones who led to the identification of influencers, however they were not directly involved in the design and conception of this study.

2.7 Ethical Clearance

Verbal explanation of the research purpose was given prior to conducting the interviews. All participants gave verbal consent over the phone for the recording and transcription of interviews which was documented on an informed consent form. The Ethical approval was obtained from

For peer review only - http://bmjopen.bmj.com/site/about/guidelines.xhtml

the College of Medicine Research & Ethics Committee (COMREC) and Medical Research Council of Zimbabwe (MRCZ/A/2683).

3. Results

Two main broad insights were drawn from thematic analysis i.e., key needs and wider contextual factors. The key needs of young PLHIV are empathy, support and connection with ART whilst the wider contextual factors with impact in their treatment journeys are structural challenges and cultural hooks.

3.1 Key needs:

Fear of shame and humiliation can be significant barriers to disclosure and treatment. The fear pre-disposes the PLHIV to the need for empathy, support and connection with ART.

3.1.1. Empathy

There was disconnect between how HCPs and PLHIV view the perceptions of the disease in the community, which can act as a barrier to connection between messenger and patient. HCPs are an important messenger, however there can sometimes be a gap in understanding or lack of connection which can mean their messages are less credible to PLHIV.

"Life here is a bit difficult if people discover that you take medicine they either discriminate you or talk behind your back, so we don't disclose to people." - PLHIV, Malawi.

"Gone are the days when it was a taboo to talk about HIV but nowadays it's now common and people accept all the messages they hear about HIV however it differs from culture to culture but generally people have accepted that HIV exists." – ART Nurse and midwife, Zimbabwe

Although HCPs say they treat PLHIV like 'anybody else' some PLHIV face discrimination in the community. For some, HIV status is not openly disclosed to the wider community as it can impact relationships.

"When it comes to professionals I guess they keep and maintain work ethics and professionalism, they treat us like any other person, they are supportive and encouraging compared to community at large." - PLHIV, Zimbabwe.

For peer review only - http://bmjopen.bmj.com/site/about/guidelines.xhtml

PLHIV often fear the worst and they often worry more about "what will people think?" rather than "what can I do to get better?" PLHIV expressed fear of judgement when telling a partner in case they were not accepting of their status, and not wanting to be seen taking medication and fear of rejection by the community.

Most of the PLHIV were more fearful of judgement as they do not want to be seen as promiscuous.

"People are so judgemental to the extent that they would say she got it through promiscuity" - PLHIV, Zimbabwe.

Initiating treatment at diagnosis is not always optimal because PLHIV are catastrophizing whilst nurses are providing a rational 'treatment seminar'. Test and Treat means that most patients are initiated to treatment straight away, but this is often not the best time to learn about treatment benefits and the importance of adherence as a patient's mental health (shock, numbness, fear, 'fight or flight', denial) makes it difficult to focus on information.

"It gave me a lot of stress, I didn't have peace and I was scared, should I tell my mom, should I start medication or I shouldn't start because of the rumours that the drugs deform people so I used to have a lot of doubts and it took me time to accept it, although at the hospital they said they weren't going to give me a chance to think about it, they said I had to start the very same day"- PLHIV, Malawi.

Taking treatment is a sign of HIV-positive status so therefore PLHIV need to hide their medication, however treatment can also be a reliever enabling the patient to show no signs of infection. PLHIV do not want members of the community knowing their status, so they lead a 'double life' to prevent discrimination.

"I feel like I am not different from others because I have no signs that shows that I am infected with the virus. At the same time this virus distanced me from my friends that I used to associate

For peer review only - http://bmjopen.bmj.com/site/about/guidelines.xhtml

with. It changed my entire lifestyle because at the present moment I am living in a lie because I don't want people to know that I am infected." - PLHIV, Zimbabwe.

Religious influence plays a major role in people's lives and the perception of PLHIV within the community. Some religious communities are reluctant to talk about HIV which may contribute to preconceptions towards the disease and further prevent disclosure or adherence. However, as HIV is not regularly spoken about in church, PLHIV fear that they will not receive the empathy they need to pull back from catastrophe in the church, nor does the church help overcome a major hurdle- spreading the message that all people including those with HIV are worthy of love equally under God.

"Many people believe that when one is HIV positive that person was very promiscuous, that is why people prefer to make it a secret about their status they do not want to be judged. Even in the community and some church organizations they do not want to talk about the virus. "- ART Nurse, Zimbabwe.

For some, ARVs are a constant reminder of lost freedom whereas for others it is liberating.
"I always think that if not for this disease, I could not have been taking this medicine and I could have been a free person." – PLHIV, Malawi

"Living with HIV doesn't mean you are dying or you are disabled it's just a condition which you can live with, and doesn't affect your lifestyle as long you take treatment as prescribed."- PLHIV, Zimbabwe.

3.1.2. Support

Often PLHIV are in shock when they receive their diagnosis, and the patients need support to accept their status to begin treatment.

"When one gets tested they are given some shallow counselling which is superficial, and start treatment, the treatment becomes very difficult because the person will not have understood, and they are still in shock maybe because they were not expecting that result."- ART Nurse & Midwife, Zimbabwe

For peer review only - http://bmjopen.bmj.com/site/about/guidelines.xhtml

Helping people accept their status is a key challenge, the sooner people accept their status the more likely they are to adhere and move on with their life.

"I was worried because what I did not expect in my life has happened. But through meeting my good friends with good counselling things started changing"- PLHIV, Malawi.

Many are concerned about disclosing to their partner and family, but when they have a positive response (most do), this makes living with the disease much easier as they have support from people around them.

"I want to thank my young sister for support and encouragement she gave me, as young as she was, she was my pillar of strength even at a time when I wanted to quit, stood with me through out"- PLHIV, Zimbabwe.

Depression and anxiety can be a significant disruptor but there is little mental health support. Treating the PLHIV holistically beyond suppressing the HIV virus alone is important to maintain adherence.

"I was depressed because of my HIV status, it stressed me a lot. Even now when I think about it deeply it still stresses me"- PLHIV, Zimbabwe.

3.1.3 Connection with ART

HCPs felt that there was good treatment literacy in both Zimbabwe and Malawi. They demonstrated significant HIV knowledge and were confident communicating messages to patients. Gaps noted by HCPs were that some PLHIV struggling with adherence tended to do so because they did not understand disease severity or benefits of medication. Knowledge of viral suppression varies across countries, but it is a motivating message. Although, there is a good basic level of knowledge around treatment benefits across all PLHIV.

For peer review only - http://bmjopen.bmj.com/site/about/guidelines.xhtml

HCPs in Zimbabwe did not widely communicate information about viral suppression. PLHIV clearly communicated the benefits of treatment and what motivated them to adhere (being healthy, controlling the virus, living a normal life, not looking like they have HIV to the community) but there were very few mentions of viral suppression or reduction in viral load.

In Malawi, some HCPs communicated information about viral suppression to PLHIV and PLHIV had a reasonable understanding of the concept. They talked about the fact that you could reach a point where the virus is not evident in your bloodstream and know that this means you will not transfer the virus to your sexual partner or child.

HCPs saw the effectiveness and impact that understanding viral suppression could have on driving behaviour-change and adherence to treatment. Especially when PLHIV are able to link viral suppression to feeling better, they become more motivated to adhere to treatment.

"They get motivated…if we tell a client that your levels are undetectable and if you continue taking medication like that you will have a long time before getting sick. Once a client hears that, they get motivated and make promises that they will maintain taking medication properly." – Clinical officer, Malawi.

3.2 Contextual Factors.

3.2.1 Structural Challenges

Poverty-related challenges remain a central barrier to adherence and the COVID-19 pandemic made the situation worse (Figure 1). PLHIV cannot take their medication if they do not have enough food to eat, even if they are motivated to do so. Sourcing food becomes core priority and HIV care is down-prioritized. COVID-19 has exacerbated existing financial challenges.

"These people must be a priority because it can be anyone, you mother, brother sister or friend. These people need care so that they do not affect others. During budget allocation the government must prioritise the Health sector and avail funds towards their welfare and resources as first preferences. I believe everyone has a role to play in the care of PLHIV."- Influencer, (maternal figure), Zimbabwe

For peer review only - http://bmjopen.bmj.com/site/about/guidelines.xhtml

Some PLHIVs were not able to afford transport to the clinics leading to missed appointments, which affected treatment schedule. This was a particular challenge in rural areas and mentioned more in Malawi.

"It is difficult when patients miss appointments and don't take their medication as their viral load gets higher and you have to spend more time educating them about taking treatment properly." – Client Officer, Malawi

There is also lack of resources in health facilities. Most health facilities do not have enough staff, meaning that less time is spent educating patients. Resource challenges include electricity blackouts, inconsistent condom supply, and occasional low stock of medications.

"It's a challenge because sometimes the ART may not be available, sometimes even the testing kits are not available, and samples are failing to go to the lab on time, end up getting stale due to lack of resources. Initially, we are supposed to retest and do Cd4 counts tests every now and then to know the patient's viral load, but unfortunately we are failing to do that. We watch people deteriorate because we have nothing to help them with."- ART Nurse, Zimbabwe

I could have recommended that they give us more nurses so that people get helped faster."- ART Nurse, Malawi

PLHIV stated that overly technical language - information from HCP may not be understood, unless explained in terms which are relatable. Some PLHIV struggle to read information leaflets, posters, and other written communication about HIV because they are too technical.

3.2.2 Cultural Hooks

People in Malawi and Zimbabwe are diverse with different hobbies and interests. There is no "one size fits all" approach and maximising campaign reach will likely need a multifaceted approach.

Young PLHIV may struggle with their disease but still find ways to have fun and live life.

For peer review only - http://bmjopen.bmj.com/site/about/guidelines.xhtml

They have identified female role models who represent independence, open-mindedness, and progress. There is a need to show PLHIV that they too can be educated, independent, and valued members of society – just like their role models. These role models embody PLHIV's aspirations and dreams, which are focused on "thriving" – becoming independent, providers, and being respected by their families and communities.

I think this must be done by everyone because people trust different people, it should be an issue discussed at household level, community, all HCPs, NGOs and social media – influencer (trainer) – Zimbabwe

Some people think HIV ended and are becoming careless. So it's important that we are not quiet, we have to do awareness campaigns, encouraging people, reminding them about HIV and its dangers. – Influencer (Religious leader) Malawi.

5. Discussion

This study provided some foundational insights into the current state of awareness of viral suppression and treatment as prevention, including the awareness and motivations of PLHIV, their influencers, and healthcare providers. The key themes demonstrated that there is a need for empathy, and for attention to be paid to wider contextual factors in communications campaigns.

Early life experiences of HIV are scary and/or traumatic. Associations include loved ones being 'taken away' and not returning, seeing people in the community who are sick & frail whilst people say they have AIDS as a catch all for any serious illness (18, 19). For healthcare workers specifically ART nurses, treating HIV might feel like a routine but for every individual PLHIV, it represents a profound change in their lives often characterised by fear, shock, sorrow, loss and low self-worth (20, 21). Our study revealed a disconnect between how HCPs and PLHIV view the perceptions of the disease in the community which can act as a barrier towards connection between messenger and patient. Focusing too heavily on educating and getting PLHIV onto treatment can mean that HCPs fail to meet the emotional needs of PLHIV, leaving them feeling even more overwhelmed and deflated.

For peer review only - http://bmjopen.bmj.com/site/about/guidelines.xhtml

There is a need to create an opportunity for the HCP-PLHIV interaction to become more transformational, rather than transactional. HCPs tend to be focused on educating to make sure PLHIV are well informed on HIV; encouraging disclosure; tracing contacts and adherence to treatment. HCPs see their role primarily as educators, secondary comes listening. On the other hand PLHIV have a lot to process in terms of diagnosis; what implications this has for their life and those around them and the new information on HIV and care.

HCPs should be orientated on the fact that rationalities regarding treatment-taking extend beyond the biomedical realm, requiring adjustments to sense of self and identity, and decision-making that is situated and socially embedded (21-23). However, in many clinical centres, standards and guidelines surrounding HIV treatment communication may be absent or lack specificity, which is a hindrance (23). HCPs can be trained to understand the types of support different patients need at different times. Treatment literacy campaigns should also consider how to still resonate with people when they are going through tough times.

It is important to understand that some PLHIV 'catastrophize' every potential disclosure of their status, particularly when out of their control. In times of hardship, people in Malawi and Zimbabwe turn to the church as a message of hope and comfort for a better future. HIV is a traumatic experience for PLHIV and they can feel like they have committed a sin. As HIV is not spoken about at a community level, they are scared that they will be shunned by all.

HCPs are already using a range of communication tools to convey viral suppression and treatment benefits in an accessible way. This was mirrored by PLHIV when expressing their knowledge of HIV. These tools could be used to scale-up the viral suppression message and reach a point where it's well recognized and understood by not just PLHIV, but the wider community too. Studies conducted in South Africa, Malawi and Kenya among health providers reported mixed attitudes about treatment as prevention although most were supportive (24-27). In Malawi, HIV care providers and program stakeholders expressed concerns related to: equating 'undetectable' with 'healed', which may impact adherence negatively, and to a potential increase in promiscuity and HIV re-infection (25). Kenyan health providers reported fears that telling patients undetectable viral loads lead to other risk behaviours, or that consequent HIV transmission would be blamed on them (26).

For peer review only - http://bmjopen.bmj.com/site/about/guidelines.xhtml

Busy clinics can mean less time spent with PLHIV and can result in gaps of education. Patients who are defaulting tend to do so because they do not understand disease severity or benefits of medication. PLHIV may still be in denial stage at the time of diagnosis and treatment. Patients are not in the right frame of mind to absorb key messages about benefits and the importance of adherence at initial treatment stage. Our findings confirm the health systems barriers such as limited and unprofessional providers at healthcare facilities, un-availability of drugs, and long travel distance that have been identified by other researchers (23, 28-31).

Strengths and Limitations of the study

We obtained a rich and nuanced appreciation of the current state of awareness of viral suppression and treatment as prevention, including the awareness and motivations as we sampled from a range of PLHIV, their influencers, and healthcare providers. The depth of inquiry allowed to identify potential areas of interest were literacy and awareness of treatment; treatment journey and ideas around bringing a community approach to supporting PLHIV with treatment. These issues were also considered in depth through a unique lens of influencers which were sampled from a variety of positions within the communities to allow a broad review.

For peer review only

Caution should be exercised when interpreting the results of this exploratory analysis due to small sample size and limited breadth of influencers sampled. Several campaigns have reinforced how people ‘should’ feel about PLHIV hence HCPs and influencers may have answered questions based on what they think is ‘correct’ or ‘acceptable’, rather than their true thoughts or beliefs which may be undesirable. The insights of the study is likely to contribute to the development of campaigns across sub-Saharan Africa, and creating of messaging that can go beyond the medical and PLHIV living a longer life.

6. Conclusion

Treatment literacy campaigns should also consider how to still resonate with people when they are going through tough times. Currently, the relationship between nurses and PLHIV can appear to be transactional. Some HCPs are hesitant to speak about viral suppression denoting the end of onward transmission risk. There is a need to show PLHIV that they too can be independent, and valued members of society. Through learning about the community (incl. PLHIV), there are more chances of communicating in a way that resonates.

For peer review only - http://bmjopen.bmj.com/site/about/guidelines.xhtml

Authors' contributions

SGM and SS were responsible for conceptualization, formal analysis, supervision, funding acquisition, validation, investigation and writing the original draft. CG, and RN contributed in resources mobilization, investigation, and project administration. PM, KC and NH contributed in the conceptualization, resources mobilization, funding acquisition, project administration. All authors read and approved the final manuscript.

Availability of Data

This study used data obtained from human participants. The dataset (anonymized survey responses) is owned by the governments of Malawi and Zimbabwe.

Competing interests

None declared.

Acknowledgements

This work was supported with leadership by the Ministry of Health and Population in Malawi and in Ministry of Health and Child Care (MoHCC) in Zimbabwe. We thank our partners Population Services International in Malawi and Zimbabwe for collaborating on the implementation of this research.

Funding Statement

This study was supported by the Flip the Script Project funded by the Johnson & Johnson and Bill & Melinda Gates Foundation. The funders were involved in the study design, but had no role in data collection and interpretation, or the decision to submit the work for publication.

For peer review only

References

1. UNAIDS. 90-90-90: An ambitious target to help end the AIDS epidemic. Geneva; 2014. Available from https://www.unaids.org/sites/default/files/media_asset/GARPR_2014_guidelines_en_0.pdf
2. UNAIDS. Political Declaration on HIV and AIDS: On the Fast Track to Accelerating the Fight against HIV and to Ending the AIDS Epidemic by 2030. Geneva; 2016. Available from https://www.unaids.org/sites/default/files/20160608_PS_HLM_Political_Declaration_final.pdf
3. UNAIDS. Understanding Fast Track: Accelerating Action to end the AIDS pandemic by 2030 2015.Available from https://www.pedaids.org/2014/11/20/unaids-issues-new-fast-track-strategy-to-end-aids-by-2030/
4. UNAIDS. 2025 AIDS TARGETS Second face-to-face meeting of the Steering Committee on Target Setting, Impact, and Resource Needs. 2019. Available from https://www.unaids.org/sites/default/files/Sep2019_2025targets-2ndStComMeeting-Report_en.pdf
5. UNAIDS. UNAIDS Fact Sheet:Preliminary UNAIDS 2021 epidemiological estimates. New York, USA. ; 2021.Available from https://www.unaids.org/sites/default/files/media_asset/UNAIDS_FactSheet_en.pdf
6. UNAIDS Data: East and Southern Africa 90-90-90 progress [Internet]. 2021. Available from: https://esahub.unaids.org/.
7. AIDSinfo. Global data on HIV epidemiology and response. 2021.Available from https://www.unaids.org/en/resources/fact-sheet
8. Diepstra K, Lu H, McManus KA, Rogawski McQuade ET, Rhodes AG, Westreich D. What we talk about when we talk about durable viral suppression. AIDS (London, England). 2020;34(11):1683-6.
9. Eisinger RW, Dieffenbach CW, Fauci AS. HIV Viral Load and Transmissibility of HIV Infection: Undetectable Equals Untransmittable. Jama. 2019;321(5):451-2.
10. U=U taking off in 2017 [press release]. LANCET2017.Available from https://www.thelancet.com/journals/lanhiv/article/PIIS2352-3018(17)30183-2/fulltext#:~:text=The%20fact%20that%20people%20infected,evidence%20since%20the%20early%202000s.
11. U=U Consensus Statement [press release]. 2021.
12. Bhatt SJ, Douglas N. Undetectable equals untransmittable (U = U): implications for preconception counseling for human immunodeficiency virus serodiscordant couples. American journal of obstetrics and gynecology. 2020;222(1):53.e1-.e4.
13. UNAIDS. Combination HIV Prevention: Tailoring and Coordinating Biomedical, Behavioural and Structural Strategies to Reduce New HIV Infections. Geneva2010. Available from https://www.unaids.org/sites/default/files/media_asset/JC2007_Combination_Prevention_paper_en_0.pdf
14. Young Holt B, Dellplain L, Creinin MD, Peine KJ, Romano J, Hemmerling A. A strategic action framework for multipurpose prevention technologies combining contraceptive hormones and antiretroviral drugs to prevent pregnancy and HIV. Eur J Contracept Reprod Health Care. 2018;23(5):326-34.

For peer review only - http://bmjopen.bmj.com/site/about/guidelines.xhtml

15. Schaefer R, Gregson S, Fearon E, Hensen B, Hallett TB, Hargreaves JR. HIV prevention cascades: a unifying framework to replicate the successes of treatment cascades. The Lancet HIV. 2019;6(1):e60-e6.
16. UNAIDS. Prevention Gap Report. 2016. Available from https://www.unaids.org/sites/default/files/media_asset/2016-prevention-gap-report_en.pdf
17. Baral S, Rao A, Sullivan P, Phaswana-Mafuya N, Diouf D, Millett G, et al. The disconnect between individual-level and population-level HIV prevention benefits of antiretroviral treatment. Lancet HIV. 2019;6(9):e632-e8.
18. Keuroghlian AS, Kamen CS, Neri E, Lee S, Liu R, Gore-Felton C. Trauma, dissociation, and antiretroviral adherence among persons living with HIV/AIDS. J Psychiatr Res. 2011;45(7):942-8.
19. Seedat S. Interventions to Improve Psychological Functioning and Health Outcomes of HIV-Infected Individuals with a History of Trauma or PTSD. Current HIV/AIDS Reports. 2012;9(4):344-50.
20. Horter S, Thabede Z, Dlamini V, Bernays S, Stringer B, Mazibuko S, et al. "Life is so easy on ART, once you accept it": Acceptance, denial and linkage to HIV care in Shiselweni, Swaziland. Social science & medicine. 2017;176:52-9.
21. Horter S, Seeley J, Bernays S, Kerschberger B, Lukhele N, Wringe A. Dissonance of Choice: Biomedical and Lived Perspectives on HIV Treatment-Taking. Med Anthropol. 2020;39(8):675-88.
22. Calabrese SK, Mayer KH, Marcus JL. Prioritising pleasure and correcting misinformation in the era of U=U. Lancet HIV. 2021;8(3):e175-e80.
23. Calabrese SK, Mayer KH. Stigma impedes HIV prevention by stifling patient-provider communication about U = U. Journal of the International AIDS Society. 2020;23(7):e25559.
24. Zhou A. The uncertainty of treatment: Women's use of HIV treatment as prevention in Malawi. Social science & medicine. 2016;158:52-60.
25. Berner-Rodoreda A, Dambe R, Ngwira E, Neuhann F, Bärnighausen T, S P. U=U - Catalyst or barrier for men‘s engagement in ART? Stakeholder views from Blantyre, Malawi. AIDS 20202020.
26. Ngure K, Ongolly F, Dolla A, Awour M, Mugwanya KK, Irungu E, et al. "I just believe there is a risk" understanding of undetectable equals untransmissible (U = U) among health providers and HIV-negative partners in serodiscordant relationships in Kenya. Journal of the International AIDS Society. 2020;23(3):e25466.
27. Lippman SA, West R, Gómez-Olivé FX, Leslie HH, Twine R, Gottert A, et al. Treatment as Prevention-Provider Knowledge and Counseling Lag Behind Global Campaigns. Journal of acquired immune deficiency syndromes (1999). 2020;83(2):e9-e12.
28. Naanyu V, Ruff J, Goodrich S, Spira T, Bateganya M, Toroitich-Ruto C, et al. Qualitative exploration of perceived benefits of care and barriers influencing HIV care in trans Nzoia, Kenya. BMC health services research. 2020;20(1):355.
29. Croome N, Ahluwalia M, Hughes LD, Abas M. Patient-reported barriers and facilitators to antiretroviral adherence in sub-Saharan Africa. AIDS (London, England). 2017;31(7):995-1007.
30. Kagee AR, R.H.; Berkman, A.; Hoffman, S.; Campos, L.; Swartz, L. . Structural barriers to ART adherence in Southern Africa: Challenges and potential ways forward. . Global public health. 2011;6:83-97.

For peer review only - http://bmjopen.bmj.com/site/about/guidelines.xhtml

31. Lockman S, Holme MP, Makhema J, Bachanas P, Moore J, Wirth KE, et al. Implementation of Universal HIV Testing and Treatment to Reduce HIV Incidence in Botswana: the Ya Tsie Study. Curr HIV/AIDS Rep. 2020;17(5):478-86.

For peer review only

Figure 1:

Legend – Structural challenges

For peer review only - http://bmjopen.bmj.com/site/about/guidelines.xhtml

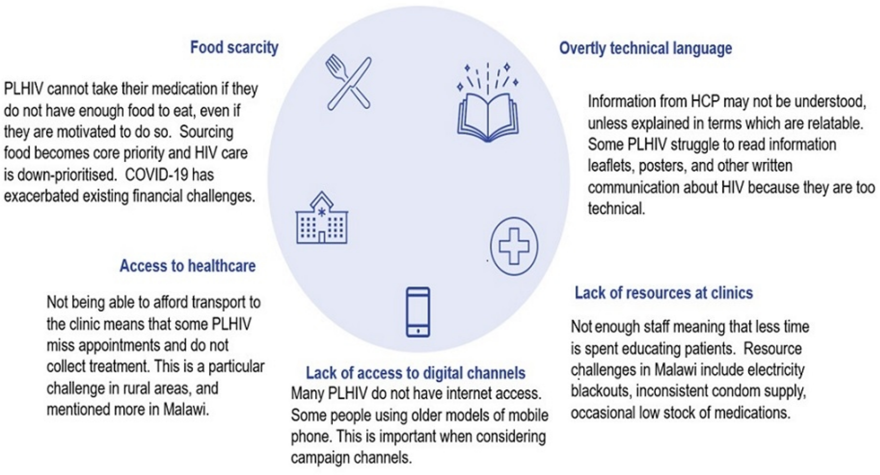

Figure 1: Structural challenges to ART adherence

Structural challenges to ART adherence

274x154mm (96 x 96 DPI)

BMJ Open

"ARVs are a constant reminder of lost freedom whereas for others they are liberating": Understanding the Treatment Narrative among People Living with HIV (PLHIV) in Malawi and Zimbabwe - A qualitative study

Journal:	*BMJ Open*
Manuscript ID	bmjopen-2022-063138.R2
Article Type:	Original research
Date Submitted by the Author:	22-Mar-2023

Complete List of Authors:	Gumede-Moyo, Sehlulekile; Ipsos MORI UK Ltd, Healthcare; London School of Hygiene & Tropical Medicine, Epidemiology and Population Health<br>Sharma, Sunny ; Ipsos MORI UK Ltd<br>Gwanzura, Clorata; Ministry of Health and Child Care<br>Nyirenda, Rose; Ministry of Health and Population Malawi<br>Mkandawire, Philip ; Population Services International<br>Chatora, Kumbirai ; Population Services International<br>Hasen, Nina; Population Services International, HIV an TB

**Primary Subject Heading**:	Evidence based practice
Secondary Subject Heading:	Epidemiology, Global health, Health services research, HIV/AIDS, Qualitative research
Keywords:	PUBLIC HEALTH, QUALITATIVE RESEARCH, HIV & AIDS < INFECTIOUS DISEASES

SCHOLARONE™ Manuscripts

I, the Submitting Author has the right to grant and does grant on behalf of all authors of the Work (as defined in the below author licence), an exclusive licence and/or a non-exclusive licence for contributions from authors who are: i) UK Crown employees; ii) where BMJ has agreed a CC-BY licence shall apply, and/or iii) in accordance with the terms applicable for US Federal Government officers or employees acting as part of their official duties; on a worldwide, perpetual, irrevocable, royalty-free basis to BMJ Publishing Group Ltd ("BMJ") its licensees and where the relevant Journal is co-owned by BMJ to the co-owners of the Journal, to publish the Work in this journal and any other BMJ products and to exploit all rights, as set out in our licence.

The Submitting Author accepts and understands that any supply made under these terms is made by BMJ to the Submitting Author unless you are acting as an employee on behalf of your employer or a postgraduate student of an affiliated institution which is paying any applicable article publishing charge ("APC") for Open Access articles. Where the Submitting Author wishes to make the Work available on an Open Access basis (and intends to pay the relevant APC), the terms of reuse of such Open Access shall be governed by a Creative Commons licence – details of these licences and which Creative Commons licence will apply to this Work are set out in our licence referred to above.

Other than as permitted in any relevant BMJ Author's Self Archiving Policies, I confirm this Work has not been accepted for publication elsewhere, is not being considered for publication elsewhere and does not duplicate material already published. I confirm all authors consent to publication of this Work and authorise the granting of this licence.

For peer review only

‘*ARVs are a constant reminder of lost freedom whereas for others they are liberating*’: Understanding the Treatment Narrative among People Living with HIV (PLHIV) in Malawi and Zimbabwe. A qualitative study.

Sehlulekile Gumede-Moyo [1,2§]; Sunny Sharma[1]; Clorata Gwanzura[3]; Rose Nyirenda[4]; Philip Mkandawire[5]; Kumbirai Chatora [6]and Nina Hasen[7]

1. Ipsos Healthcare, London, United Kingdom,

2. Department of Population Health, Faculty of Epidemiology and Population Health, London School of Hygiene and Tropical Medicine, London, UK

3. Department of AIDS & TB, Ministry of Health and Child Care, Harare, Zimbabwe

4. Department of HIV & AIDS, Ministry of Health and Population, Malawi

5. Population Services International, Malawi.

6. Population Services International, Zimbabwe.

7. Population Service International Global.

[§]Corresponding author: Sehlulekile Gumede-Moyo, PhD, MPH, MBA. (seh.sokhela@gmail.com)

Key words: PLHIV; viral suppression; empathy; support; connection; campaigns.

For peer review only - http://bmjopen.bmj.com/site/about/guidelines.xhtml

Abstract

Objective: The aim of the research was to understand what drives and motivates young women living with HIV in their treatment journeys, as well as their key influencers. The findings will assist in appreciating their obstacles to treatment and constructing the most effective ways to convey fresh messages to them. These insights will contribute to the messaging developed for a campaign across sub-Saharan Africa, but primarily Malawi and Zimbabwe.

Design: This was a qualitative study conducted in order to build an understanding of unknown parts of the HIV treatment journey through in-depth interviews. A hybrid approach was used to conduct thematic analysis.

Setting and Participants: Study participants were HIV-positive women, their influencers (e.g. parents/ partners/ siblings/ aunts and uncles/ religious leaders etc.), and healthcare providers from a range of regions in Zimbabwe and Malawi.

Results: A total of 26 young PLHIV, 29 HCPs, and 24 influencers such as maternal figures in the community were interviewed. Two main broad insights were drawn i.e., key needs and wider contextual factors. The key needs of young PLHIV to stay on treatment were empathy, support, and emotional connection with ART whilst the wider contextual factors impacting their treatment journeys were structural challenges and cultural reference points. Fear of shame and humiliation can also be significant barriers to disclosure and treatment. The fear pre-disposes the PLHIV to the need for empathy, support, and connection with ART. Mental health and anxiety appear to be co-morbid with HIV. Some PLHIV have a small and limited support network leading to very few people living openly with HIV. There is no "one size fits all" approach and maximizing campaign reach will likely need a multifaceted approach.

Conclusion: Currently the relationship between nurses and PLHIV can appear to be transactional. Through learning about the community (including PLHIV), there are more chances of communicating in a way that resonates.

For peer review only - http://bmjopen.bmj.com/site/about/guidelines.xhtml

Strengths

- The participants of the study were sampled from a range of PLHIV, their influencers, and healthcare providers.
- The depth of inquiry allowed to identify potential areas of interest were literacy and awareness of treatment.

Limitations

- Several campaigns reinforcing how people 'should' feel about PLHIV may have influenced the research participants to respond in a social desirable manner.

For peer review only

For peer review only - http://bmjopen.bmj.com/site/about/guidelines.xhtml

1. Introduction

The UNAIDS 95-95-95 targets aim to achieve a 95% level of diagnosis of people living with HIV (PLHIV) 95% of those diagnosed on antiretroviral therapy (ART), and 95% of those on ART virally suppressed by 2025 (1-4). In 2020, of all PLHIV globally, 84% knew their status, 73% (57–88%) were accessing treatment and 66% (53–79%) were virally suppressed (5). East and Southern Africa are the regions most affected by HIV are home to the largest numbers of PLHIV (6). While the number of PLHIV in East and Southern Africa continues to increase, access to ART is increasing as well (7). Young people, especially women and key populations, continue to have a disproportionately low level of viral suppression (4). One goal of HIV care and treatment is achieving an undetectable viral load as it confers benefits at both the individual and public health-level (8, 9). There is strong evidence that supports that PLHIV cannot sexually transmit HIV to others when their viral load is suppressed to 200 HIV RNA copies/mL or lower, and they have been adherent to ART for at least 6 months (8, 10-12).

UNAIDS have called for combined approaches to scale up HIV prevention, to reinvigorate the global response and make a sustained impact on global HIV incidence rates (13). Combining separate strategies for different indications into singular prevention products can reduce the stigma around HIV and STI prevention, improve acceptability of and adherence to more convenient products, and be more cost-effective by addressing overlapping risks (14, 15). All combination prevention programmes require a strong community empowerment element and specific efforts to address legal and policy barriers, as well as the strengthening of health and social protection systems, plus actions to address gender inequality, stigma and discrimination (16, 17).

The majority of the existing HIV treatment campaigns in sub-Saharan Africa (SSA) are one-dimensional when it comes to the benefits of treatment, the fact that treatment extends life being the key benefit, rather than also focusing on other factors which could resonate more personally with individual PLHIV. The aim of the research was to understand what drives and motivates young women living with HIV, as well as their key influencers. The findings will assist in appreciating their obstacles to treatment and constructing the most effective ways to convey fresh messages to them.

For peer review only - http://bmjopen.bmj.com/site/about/guidelines.xhtml

2. Methods

2.1 Study Design

This was a qualitative study conducted in order to build an understanding of unknown parts of the HIV treatment journey through in-depth interviews with healthcare workers, young people living with HIV and their influencers (e.g. parents/ partners/ siblings/ aunts and uncles/ religious leaders etc.). The potential areas of interest were literacy and awareness of treatment; understanding their treatment journey (what is communicated to them about treatment, how well is it understood, what is compelling (if anything), barriers) and ideas around bringing a community approach to supporting PLHIV with treatment.

2.2 Study Setting

The research seek to deliver foundational insights on the current state of awareness of viral suppression and treatment as prevention, including the awareness and motivations of PLHIV, their influencers, and healthcare providers to stick with, counsel, or support taking their medicine every day. These insights will contribute to the messaging developed for a campaign across sub-Saharan Africa, but primarily Malawi and Zimbabwe that will aim to shift perspectives of PLHIV to take action based on the new concept / narrative of treatment literacy.

2.3 Sample recruitment

Our main study participants were HIV positive women, their influencers and healthcare providers from a range of regions in Zimbabwe and Malawi. The breakdown of study participants are indicated in Supplementary Table 1. Eligibility criteria and recruitment procedures are described below. Young HIV positive women (18-35 years) were targeted as they have disproportionately low levels of viral suppression based on UNAIDS reports (4-6) .

2.3.1 PLHIV

The participants' names were retrieved from the Population Services International (PSI)-assisted ART clinic's database. The eligibility for the PLHIV was for one to be: 18-35 years old PLHIV (diagnosed at least 12 months ago); and not pregnant or breastfeeding. Quotas were set to recruit 1/3 of participants on treatment with good adherence, and 2/3 not taking treatment (non-adherence); a mix of respondents who had been on treatment for long and shorter periods of time, and a mix of those who were virally suppressed and those who were not.

For peer review only - http://bmjopen.bmj.com/site/about/guidelines.xhtml

2.3.2 HCPs

Eligibility criteria for HCP recruitment included having at least 3 years' experience in their current role, spending at least 50% of their time working in HIV care, and interacting with at least 5 HIV+ women (aged 18-35) each day. HCPs worked at a variety of sites including public and private facilities.

2.3.3 Influencers

For this study an influencer was defined as someone who has an impact on PLHIV, either as part of their treatment journey or friends/relatives. The influencers were recruited after initial analysis of the PLHIV transcripts which determined who they were. These were maternal/grandmother figures, workshop leaders and WhatsApp group leaders. Workshops are support groups where PLHIV can meet to discuss concerns, ask questions, and receive peer support, workshop trainers are individuals that run these workshops. WhatsApp group chats are set up amongst communities to act as peer support networks consisting of PLHIV and their family/friends. WhatsApp group leaders are individuals that run these group chats in the communities. These influencers were recruited through snowballing.

2.4 Data collection

Data were collected from December 2020 to February 2021 in both countries. Semi-structured interviews were conducted by trained moderators with vast experience in qualitative data collection. Guides were produced in English, and then translated to Chichewa for Malawi, and into Ndebele and Shona for Zimbabwe. Study participants were asked before beginning the interview their language preference. Pretesting of the guides was done according to procedure. Due to the developing COVID-19 pandemic, planned face-to-face interviews were replaced by in-depth telephone interviews lasting approximately 45-75 minutes. If at any point phone lines were interrupted or participants had to pause/stop the interview, the interviewers arranged to call back at a suitable time. All interviews were audio-recorded for translation and transcription. Each participant was allocated with a unique personal identifier (ID) to maintain anonymity.

review only

For peer review only - http://bmjopen.bmj.com/site/about/guidelines.xhtml

2.4 Data collection

Data were collected from December 2020 to February 2021 in both countries. Semi-structured interviews were conducted by trained moderators with vast experience in qualitative data collection. Guides were produced in English, and then translated to Chichewa for Malawi, and into Ndebele and Shona for Zimbabwe. Study participants were asked before beginning the interview their language preference. Pretesting of the guides was done according to procedure. Due to the developing COVID-19 pandemic, planned face-to-face interviews were replaced by in-depth telephone interviews lasting approximately 45-75 minutes. If at any point phone lines were interrupted or participants had to pause/stop the interview, the interviewers arranged to call back at a suitable time. All interviews were audio-recorded for translation and transcription. Each participant was allocated with a unique personal identifier (ID) to maintain anonymity.

2.5 Data analysis

Smart transcript was used to group the responses in Excel. A hybrid (i.e., inductive and deductive) approach was used to conduct thematic analysis. Four research analysts (2 per country) independently coded the transcripts, after familiarising themselves with the scripts. The coded data were then shared with field managers who had field notes as additional sources of data. In cases where there were discrepancies, all coders rechecked the transcripts and held meetings with the principal investigators and research managers until agreement was achieved. Anonymised quotes from some participants were also included in the manuscript to illustrate certain themes.

2.6 Patient and public involvement

The study participants were involved in the research recruitment, as they were the ones who led to the identification of influencers, however they were not directly involved in the design and conception of this study.

3. Results

Two main broad insights were drawn from thematic analysis i.e., key needs and wider contextual factors. The key needs of young PLHIV are empathy, support and connection with ART whilst the wider contextual factors with impact in their treatment journeys are structural challenges and cultural hooks.

For peer review only - http://bmjopen.bmj.com/site/about/guidelines.xhtml

3.1 Key needs:

Fear of shame and humiliation can be significant barriers to disclosure and treatment. The fear pre-disposes the PLHIV to the need for empathy, support and connection with ART.

3.1.1. Empathy

There was disconnect between how HCPs and PLHIV view the perceptions of the disease in the community, which can act as a barrier to connection between messenger and patient. HCPs are an important messenger, however there can sometimes be a gap in understanding or lack of connection which can mean their messages are less credible to PLHIV.

"Life here is a bit difficult if people discover that you take medicine they either discriminate you or talk behind your back, so we don't disclose to people." - PLHIV, Malawi.

"Gone are the days when it was a taboo to talk about HIV but nowadays it's now common and people accept all the messages they hear about HIV however it differs from culture to culture but generally people have accepted that HIV exists." – ART Nurse and midwife, Zimbabwe

Although HCPs say they treat PLHIV like 'anybody else' some PLHIV face discrimination in the community. For some, HIV status is not openly disclosed to the wider community as it can impact relationships.

"When it comes to professionals I guess they keep and maintain work ethics and professionalism, they treat us like any other person, they are supportive and encouraging compared to community at large." - PLHIV, Zimbabwe.

PLHIV often fear the worst and they often worry more about "what will people think?" rather than "what can I do to get better?" PLHIV expressed fear of judgement when telling a partner in case they were not accepting of their status, and not wanting to be seen taking medication and fear of rejection by the community.

Most of the PLHIV were more fearful of judgement as they do not want to be seen as promiscuous.

For peer review only

"People are so judgemental to the extent that they would say she got it through promiscuity" - PLHIV, Zimbabwe.

Initiating treatment at diagnosis is not always optimal because PLHIV are catastrophizing whilst nurses are providing a rational 'treatment seminar'. Test and Treat means that most patients are initiated to treatment straight away, but this is often not the best time to learn about treatment benefits and the importance of adherence as a patient's mental health (shock, numbness, fear, 'fight or flight', denial) makes it difficult to focus on information.

"It gave me a lot of stress, I didn't have peace and I was scared, should I tell my mom, should I start medication or I shouldn't start because of the rumours that the drugs deform people so I used to have a lot of doubts and it took me time to accept it, although at the hospital they said they weren't going to give me a chance to think about it, they said I had to start the very same day"- PLHIV, Malawi.

Taking treatment is a sign of HIV-positive status so therefore PLHIV need to hide their medication, however treatment can also be a reliever enabling the patient to show no signs of infection. PLHIV do not want members of the community knowing their status, so they lead a 'double life' to prevent discrimination.

"I feel like I am not different from others because I have no signs that shows that I am infected with the virus. At the same time this virus distanced me from my friends that I used to associate with. It changed my entire lifestyle because at the present moment I am living in a lie because I don't want people to know that I am infected." - PLHIV, Zimbabwe.

Religious influence plays a major role in people's lives and the perception of PLHIV within the community. Some religious communities are reluctant to talk about HIV which may contribute to preconceptions towards the disease and further prevent disclosure or adherence. However, as HIV is not regularly spoken about in church, PLHIV fear that they will not receive the empathy they need to pull back from catastrophe in the church, nor does the church help overcome a

For peer review only - http://bmjopen.bmj.com/site/about/guidelines.xhtml

major hurdle- spreading the message that all people including those with HIV are worthy of love equally under God.

"Many people believe that when one is HIV positive that person was very promiscuous, that is why people prefer to make it a secret about their status they do not want to be judged. Even in the community and some church organizations they do not want to talk about the virus. "- ART Nurse, Zimbabwe.

For some, ARVs are a constant reminder of lost freedom whereas for others it is liberating.
"I always think that if not for this disease, I could not have been taking this medicine and I could have been a free person." – PLHIV, Malawi

"Living with HIV doesn't mean you are dying or you are disabled it's just a condition which you can live with, and doesn't affect your lifestyle as long you take treatment as prescribed."- PLHIV, Zimbabwe.

3.1.2. Support

Often PLHIV are in shock when they receive their diagnosis, and the patients need support to accept their status to begin treatment.

"When one gets tested they are given some shallow counselling which is superficial, and start treatment, the treatment becomes very difficult because the person will not have understood, and they are still in shock maybe because they were not expecting that result."- ART Nurse & Midwife, Zimbabwe

Helping people accept their status is a key challenge, the sooner people accept their status the more likely they are to adhere and move on with their life.

"I was worried because what I did not expect in my life has happened. But through meeting my good friends with good counselling things started changing"- PLHIV, Malawi.

For peer review only - http://bmjopen.bmj.com/site/about/guidelines.xhtml

Many are concerned about disclosing to their partner and family, but when they have a positive response (most do), this makes living with the disease much easier as they have support from people around them.

"I want to thank my young sister for support and encouragement she gave me, as young as she was, she was my pillar of strength even at a time when I wanted to quit, stood with me through out"- PLHIV, Zimbabwe.

Depression and anxiety can be a significant disruptor but there is little mental health support. Treating the PLHIV holistically beyond suppressing the HIV virus alone is important to maintain adherence.

"I was depressed because of my HIV status, it stressed me a lot. Even now when I think about it deeply it still stresses me"- PLHIV, Zimbabwe.

3.1.3 Connection with ART

HCPs felt that there was good treatment literacy in both Zimbabwe and Malawi. They demonstrated significant HIV knowledge and were confident communicating messages to patients. Gaps noted by HCPs were that some PLHIV struggling with adherence tended to do so because they did not understand disease severity or benefits of medication. Knowledge of viral suppression varies across countries, but it is a motivating message. Although, there is a good basic level of knowledge around treatment benefits across all PLHIV.

HCPs in Zimbabwe did not widely communicate information about viral suppression. PLHIV clearly communicated the benefits of treatment and what motivated them to adhere (being healthy, controlling the virus, living a normal life, not looking like they have HIV to the community) but there were very few mentions of viral suppression or reduction in viral load.

In Malawi, some HCPs communicated information about viral suppression to PLHIV and PLHIV had a reasonable understanding of the concept. They talked about the fact that you could

For peer review only - http://bmjopen.bmj.com/site/about/guidelines.xhtml

reach a point where the virus is not evident in your bloodstream and know that this means you will not transfer the virus to your sexual partner or child.

HCPs saw the effectiveness and impact that understanding viral suppression could have on driving behaviour-change and adherence to treatment. Especially when PLHIV are able to link viral suppression to feeling better, they become more motivated to adhere to treatment.

"They get motivated…if we tell a client that your levels are undetectable and if you continue taking medication like that you will have a long time before getting sick. Once a client hears that, they get motivated and make promises that they will maintain taking medication properly." – Clinical officer, Malawi.

3.2 Contextual Factors.

3.2.1 Structural Challenges

Poverty-related challenges remain a central barrier to adherence and the COVID-19 pandemic made the situation worse (Figure 1). PLHIV cannot take their medication if they do not have enough food to eat, even if they are motivated to do so. Sourcing food becomes core priority and HIV care is down-prioritized. COVID-19 has exacerbated existing financial challenges.

"These people must be a priority because it can be anyone, you mother, brother sister or friend. These people need care so that they do not affect others. During budget allocation the government must prioritise the Health sector and avail funds towards their welfare and resources as first preferences. I believe everyone has a role to play in the care of PLHIV."- Influencer, (maternal figure), Zimbabwe

Some PLHIVs were not able to afford transport to the clinics leading to missed appointments, which affected treatment schedule. This was a particular challenge in rural areas and mentioned more in Malawi.

"It is difficult when patients miss appointments and don't take their medication as their viral load gets higher and you have to spend more time educating them about taking treatment properly." – Client Officer, Malawi

For peer review only - http://bmjopen.bmj.com/site/about/guidelines.xhtml

There is also lack of resources in health facilities. Most health facilities do not have enough staff, meaning that less time is spent educating patients. Resource challenges include electricity blackouts, inconsistent condom supply, and occasional low stock of medications.

"It's a challenge because sometimes the ART may not be available, sometimes even the testing kits are not available, and samples are failing to go to the lab on time, end up getting stale due to lack of resources. Initially, we are supposed to retest and do Cd4 counts tests every now and then to know the patient's viral load, but unfortunately we are failing to do that. We watch people deteriorate because we have nothing to help them with."- ART Nurse, Zimbabwe

I could have recommended that they give us more nurses so that people get helped faster."- ART Nurse, Malawi

PLHIV stated that overly technical language - information from HCP may not be understood, unless explained in terms which are relatable. Some PLHIV struggle to read information leaflets, posters, and other written communication about HIV because they are too technical.

3.2.2 Cultural Hooks

People in Malawi and Zimbabwe are diverse with different hobbies and interests. There is no "one size fits all" approach and maximising campaign reach will likely need a multifaceted approach.

Young PLHIV may struggle with their disease but still find ways to have fun and live life. They have identified female role models who represent independence, open-mindedness, and progress. There is a need to show PLHIV that they too can be educated, independent, and valued members of society – just like their role models. These role models embody PLHIV's aspirations and dreams, which are focused on "thriving" – becoming independent, providers, and being respected by their families and communities.

For peer review only - http://bmjopen.bmj.com/site/about/guidelines.xhtml

I think this must be done by everyone because people trust different people, it should be an issue discussed at household level, community, all HCPs, NGOs and social media – influencer (trainer) – Zimbabwe

Some people think HIV ended and are becoming careless. So it's important that we are not quiet, we have to do awareness campaigns, encouraging people, reminding them about HIV and its dangers. – Influencer (Religious leader) Malawi.

5. Discussion

This study provided some foundational insights into the current state of awareness of viral suppression and treatment as prevention, including the awareness and motivations of PLHIV, their influencers, and healthcare providers. The key themes demonstrated that there is a need for empathy, and for attention to be paid to wider contextual factors in communications campaigns.

Early life experiences of HIV are scary and/or traumatic. Associations include loved ones being 'taken away' and not returning, seeing people in the community who are sick & frail whilst people say they have AIDS as a catch all for any serious illness (18, 19). For healthcare workers specifically ART nurses, treating HIV might feel like a routine but for every individual PLHIV, it represents a profound change in their lives often characterised by fear, shock, sorrow, loss and low self-worth (20, 21). Our study revealed disconnect between how HCPs and PLHIV view the perceptions of the disease in the community which can act as a barrier towards connection between messenger and patient. Focusing too heavily on educating and getting PLHIV onto treatment can mean that HCPs fail to meet the emotional needs of PLHIV, leaving them feeling even more overwhelmed and deflated.

There is a need to create an opportunity for the HCP-PLHIV interaction to become more transformational, rather than transactional. HCPs tend to be focused on educating to make sure PLHIV are well informed on HIV; encouraging disclosure; tracing contacts and adherence to treatment. HCPs see their role primarily as educators, secondary comes listening. On the other hand PLHIV have a lot to process in terms of diagnosis; what implications this has for their life and those around them and the new information on HIV and care.

For peer review only - http://bmjopen.bmj.com/site/about/guidelines.xhtml

HCPs should be orientated on the fact that rationalities regarding treatment-taking extend beyond the biomedical realm, requiring adjustments to sense of self and identity, and decision-making that is situated and socially embedded (21-23). However, in many clinical centres, standards and guidelines surrounding HIV treatment communication may be absent or lack specificity, which is a hindrance (23). HCPs can be trained to understand the types of support different patients need at different times. Treatment literacy campaigns should also consider how to still resonate with people when they are going through tough times.

It is important to understand that some PLHIV ‘catastrophize’ every potential disclosure of their status, particularly when out of their control. In times of hardship, people in Malawi and Zimbabwe turn to the church as a message of hope and comfort for a better future. HIV is a traumatic experience for PLHIV and they can feel like they have committed a sin. As HIV is not spoken about at a community level, they are scared that they will be shunned by all.
HCPs are already using a range of communication tools to convey viral suppression and treatment benefits in an accessible way. This was mirrored by PLHIV when expressing their knowledge of HIV. These tools could be used to scale-up the viral suppression message and reach a point where it's well recognized and understood by not just PLHIV, but the wider community too. Studies conducted in South Africa, Malawi and Kenya among health providers reported mixed attitudes about treatment as prevention although most were supportive (24-27). In Malawi, HIV care providers and program stakeholders expressed concerns related to: equating ‘undetectable’ with ‘healed’, which may impact adherence negatively, and to a potential increase in promiscuity and HIV re-infection (25). Kenyan health providers reported fears that telling patients undetectable viral loads lead to other risk behaviours, or that consequent HIV transmission would be blamed on them (26).

Busy clinics can mean less time spent with PLHIV and can result in gaps of education. Patients who are defaulting tend to do so because they do not understand disease severity or benefits of medication. PLHIV may still be in denial stage at the time of diagnosis and treatment. Patients are not in the right frame of mind to absorb key messages about benefits and the importance of adherence at initial treatment stage. Our findings confirm the health systems barriers such as

For peer review only - http://bmjopen.bmj.com/site/about/guidelines.xhtml

limited and unprofessional providers at healthcare facilities, un-availability of drugs, and long travel distance that have been identified by other researchers (23, 28-31).

Strengths and Limitations of the study

We obtained a rich and nuanced appreciation of the current state of awareness of viral suppression and treatment as prevention, including the awareness and motivations as we sampled from a range of PLHIV, their influencers, and healthcare providers. The depth of inquiry allowed to identify potential areas of interest were literacy and awareness of treatment; treatment journey and ideas around bringing a community approach to supporting PLHIV with treatment. These issues were also considered in depth through a unique lens of influencers which were sampled from a variety of positions within the communities to allow a broad review.

Caution should be exercised when interpreting the results of this exploratory analysis due to small sample size and limited breadth of influencers sampled. Several campaigns have reinforced how people 'should' feel about PLHIV hence HCPs and influencers may have answered questions based on what they think is 'correct' or 'acceptable', rather than their true thoughts or beliefs which may be undesirable. The insights of the study is likely to contribute to the development of campaigns across sub-Saharan Africa, and creating of messaging that can go beyond the medical and PLHIV living a longer life.

6. Conclusion

Treatment literacy campaigns should also consider how to still resonate with people when they are going through tough times. Currently, the relationship between nurses and PLHIV can appear to be transactional. Some HCPs are hesitant to speak about viral suppression denoting the end of onward transmission risk. There is a need to show PLHIV that they too can be independent, and valued members of society. Through learning about the community (including PLHIV), there are more chances of communicating in a way that resonates.

Authors' contributions

SGM and SS were responsible for conceptualization, formal analysis, supervision, funding acquisition, validation, investigation and writing the original draft. CG, and RN contributed in resources mobilization, investigation, and project administration. PM, KC and NH contributed in

For peer review only - http://bmjopen.bmj.com/site/about/guidelines.xhtml

the conceptualization, resources mobilization, funding acquisition, project administration. All authors read and approved the final manuscript.

Availability of Data

This study used data obtained from human participants. The dataset (anonymized survey responses) is owned by the governments of Malawi and Zimbabwe.

Competing interests

None declared.

Acknowledgements

This work was supported with leadership by the Ministry of Health and Population in Malawi and in Ministry of Health and Child Care (MoHCC) in Zimbabwe. We thank our partners Population Services International in Malawi and Zimbabwe for collaborating on the implementation of this research.

Funding Statement

This study was supported by the Flip the Script Project funded by the Johnson & Johnson and Bill & Melinda Gates Foundation. The funders were involved in the study design, but had no role in data collection and interpretation, or the decision to submit the work for publication.

Ethics

Verbal explanation of the research purpose was given prior to conducting the interviews. All participants gave verbal consent over the phone for the recording and transcription of interviews which was documented on an informed consent form. The Ethical approval was obtained from the College of Medicine Research & Ethics Committee (COMREC) and Medical Research Council of Zimbabwe (MRCZ/A/2683).

For peer review only

References

1. UNAIDS. 90-90-90: An ambitious target to help end the AIDS epidemic. Geneva; 2014. Available from https://www.unaids.org/sites/default/files/media_asset/GARPR_2014_guidelines_en_0.pdf
2. UNAIDS. Political Declaration on HIV and AIDS: On the Fast Track to Accelerating the Fight against HIV and to Ending the AIDS Epidemic by 2030. Geneva; 2016. Available from https://www.unaids.org/sites/default/files/20160608_PS_HLM_Political_Declaration_final.pdf
3. UNAIDS. Understanding Fast Track: Accelerating Action to end the AIDS pandemic by 2030 2015.Available from https://www.pedaids.org/2014/11/20/unaids-issues-new-fast-track-strategy-to-end-aids-by-2030/
4. UNAIDS. 2025 AIDS TARGETS Second face-to-face meeting of the Steering Committee on Target Setting, Impact, and Resource Needs. 2019. Available from https://www.unaids.org/sites/default/files/Sep2019_2025targets-2ndStComMeeting-Report_en.pdf
5. UNAIDS. UNAIDS Fact Sheet:Preliminary UNAIDS 2021 epidemiological estimates. New York, USA. ; 2021.Available from https://www.unaids.org/sites/default/files/media_asset/UNAIDS_FactSheet_en.pdf
6. UNAIDS Data: East and Southern Africa 90-90-90 progress [Internet]. 2021. Available from: https://esahub.unaids.org/.
7. AIDSinfo. Global data on HIV epidemiology and response. 2021.Available from https://www.unaids.org/en/resources/fact-sheet
8. Diepstra K, Lu H, McManus KA, Rogawski McQuade ET, Rhodes AG, Westreich D. What we talk about when we talk about durable viral suppression. AIDS (London, England). 2020;34(11):1683-6.
9. Eisinger RW, Dieffenbach CW, Fauci AS. HIV Viral Load and Transmissibility of HIV Infection: Undetectable Equals Untransmittable. Jama. 2019;321(5):451-2.
10. U=U taking off in 2017 [press release]. LANCET2017.Available from https://www.thelancet.com/journals/lanhiv/article/PIIS2352-3018(17)30183-2/fulltext#:~:text=The%20fact%20that%20people%20infected,evidence%20since%20the%20early%202000s.
11. U=U Consensus Statement [press release]. 2021.
12. Bhatt SJ, Douglas N. Undetectable equals untransmittable (U = U): implications for preconception counseling for human immunodeficiency virus serodiscordant couples. American journal of obstetrics and gynecology. 2020;222(1):53.e1-.e4.
13. UNAIDS. Combination HIV Prevention: Tailoring and Coordinating Biomedical, Behavioural and Structural Strategies to Reduce New HIV Infections. Geneva2010. Available from https://www.unaids.org/sites/default/files/media_asset/JC2007_Combination_Prevention_paper_en_0.pdf
14. Young Holt B, Dellplain L, Creinin MD, Peine KJ, Romano J, Hemmerling A. A strategic action framework for multipurpose prevention technologies combining contraceptive hormones and antiretroviral drugs to prevent pregnancy and HIV. Eur J Contracept Reprod Health Care. 2018;23(5):326-34.

For peer review only - http://bmjopen.bmj.com/site/about/guidelines.xhtml

15. Schaefer R, Gregson S, Fearon E, Hensen B, Hallett TB, Hargreaves JR. HIV prevention cascades: a unifying framework to replicate the successes of treatment cascades. The Lancet HIV. 2019;6(1):e60-e6.
16. UNAIDS. Prevention Gap Report. 2016. Available from https://www.unaids.org/sites/default/files/media_asset/2016-prevention-gap-report_en.pdf
17. Baral S, Rao A, Sullivan P, Phaswana-Mafuya N, Diouf D, Millett G, et al. The disconnect between individual-level and population-level HIV prevention benefits of antiretroviral treatment. Lancet HIV. 2019;6(9):e632-e8.
18. Keuroghlian AS, Kamen CS, Neri E, Lee S, Liu R, Gore-Felton C. Trauma, dissociation, and antiretroviral adherence among persons living with HIV/AIDS. J Psychiatr Res. 2011;45(7):942-8.
19. Seedat S. Interventions to Improve Psychological Functioning and Health Outcomes of HIV-Infected Individuals with a History of Trauma or PTSD. Current HIV/AIDS Reports. 2012;9(4):344-50.
20. Horter S, Thabede Z, Dlamini V, Bernays S, Stringer B, Mazibuko S, et al. "Life is so easy on ART, once you accept it": Acceptance, denial and linkage to HIV care in Shiselweni, Swaziland. Social science & medicine. 2017;176:52-9.
21. Horter S, Seeley J, Bernays S, Kerschberger B, Lukhele N, Wringe A. Dissonance of Choice: Biomedical and Lived Perspectives on HIV Treatment-Taking. Med Anthropol. 2020;39(8):675-88.
22. Calabrese SK, Mayer KH, Marcus JL. Prioritising pleasure and correcting misinformation in the era of U=U. Lancet HIV. 2021;8(3):e175-e80.
23. Calabrese SK, Mayer KH. Stigma impedes HIV prevention by stifling patient-provider communication about U = U. Journal of the International AIDS Society. 2020;23(7):e25559.
24. Zhou A. The uncertainty of treatment: Women's use of HIV treatment as prevention in Malawi. Social science & medicine. 2016;158:52-60.
25. Berner-Rodoreda A, Dambe R, Ngwira E, Neuhann F, Bärnighausen T, S P. U=U - Catalyst or barrier for men‘s engagement in ART? Stakeholder views from Blantyre, Malawi. AIDS 20202020.
26. Ngure K, Ongolly F, Dolla A, Awour M, Mugwanya KK, Irungu E, et al. "I just believe there is a risk" understanding of undetectable equals untransmissible (U = U) among health providers and HIV-negative partners in serodiscordant relationships in Kenya. Journal of the International AIDS Society. 2020;23(3):e25466.
27. Lippman SA, West R, Gómez-Olivé FX, Leslie HH, Twine R, Gottert A, et al. Treatment as Prevention-Provider Knowledge and Counseling Lag Behind Global Campaigns. Journal of acquired immune deficiency syndromes (1999). 2020;83(2):e9-e12.
28. Naanyu V, Ruff J, Goodrich S, Spira T, Bateganya M, Toroitich-Ruto C, et al. Qualitative exploration of perceived benefits of care and barriers influencing HIV care in trans Nzoia, Kenya. BMC health services research. 2020;20(1):355.
29. Croome N, Ahluwalia M, Hughes LD, Abas M. Patient-reported barriers and facilitators to antiretroviral adherence in sub-Saharan Africa. AIDS (London, England). 2017;31(7):995-1007.
30. Kagee AR, R.H.; Berkman, A.; Hoffman, S.; Campos, L.; Swartz, L. . Structural barriers to ART adherence in Southern Africa: Challenges and potential ways forward. . Global public health. 2011;6:83-97.

For peer review only - http://bmjopen.bmj.com/site/about/guidelines.xhtml

31. Lockman S, Holme MP, Makhema J, Bachanas P, Moore J, Wirth KE, et al. Implementation of Universal HIV Testing and Treatment to Reduce HIV Incidence in Botswana: the Ya Tsie Study. Curr HIV/AIDS Rep. 2020;17(5):478-86.

For peer review only

Figure 1:

Legend – Structural challenges

For peer review only - http://bmjopen.bmj.com/site/about/guidelines.xhtml

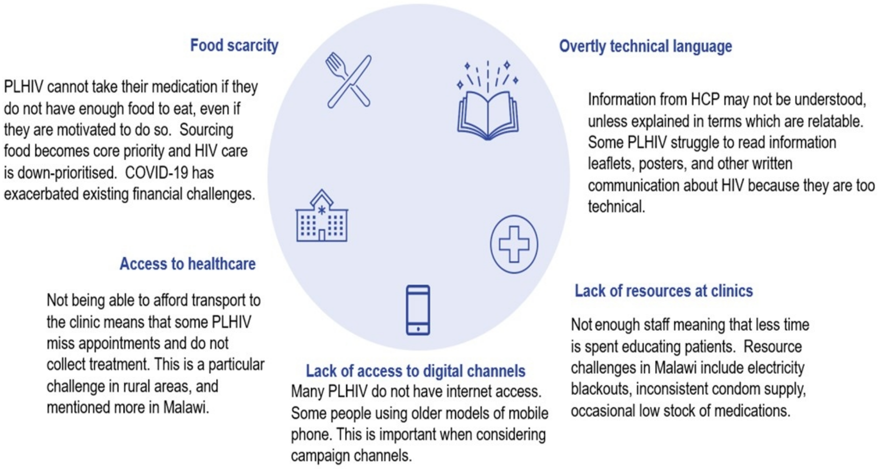

Figure 1: Structural challenges to ART adherence

Structural challenges to ART adherence

338x190mm (96 x 96 DPI)

Supplementary Table 1

Country			Zimbabwe				Malawi					Sub Total
Region			Harare	Bulawayo	Midlands	Masvingo		Lilongwe	Blantyre	Thylo	Mangochi	
PLHIV	Age group	18-21	2	1	0	1		1	1	0	0	6
		22-25	0	0	1	0		0	2	1	0	4
		26-30	0	2	1	1		0	0	0	0	4
		31-35	1	0	1	1		2	3	1	3	12
		**Total**										**26**
HCPS	Gender	Female	2	3	2	1		2	2	3	2	17
		Male	1	0	1	2		1	3	3	1	12
	Job Title	Peer Counsellor	1	1	1	1	Expert Client	2	1	2	1	10
		Integrated Care Nurses		1		1	Clinical Officer	1	2	2	1	8
		ART Nurse	1	1	1	1	ART Nurse	2	0	1	1	
		Sexual Reproductive Health(SRH) Nurses	1		1		HIV Testing Services (HTS) Nurse	0	1	0	0	3
							HTS Focus Person	0	1	0	0	1
		**Total**										**29**
Influencers	Type of Role	Maternal Figure	1	1	1	1	Maternal Figure	1	1	1	1	8
		WhatsApp Group Leaders	1	1	1	1	WhatsApp Group Leaders	1	1	1	1	8
		Workshop Coordinators	1	1	1	1	Religious Leaders/Pastors	1	1	1	1	8
		Total										**24**

For peer review only

Standards for Reporting Qualitative Research (SRQR)*

http://www.equator-network.org/reporting-guidelines/srqr/

Page/line no(s).

Title and abstract

**Title** - Concise description of the nature and topic of the study Identifying the study as qualitative or indicating the approach (e.g., ethnography, grounded theory) or data collection methods (e.g., interview, focus group) is recommended	Page 1 Lines 1-3
**Abstract** - Summary of key elements of the study using the abstract format of the intended publication; typically includes background, purpose, methods, results, and conclusions	Page 2 Lines 2-30

Introduction

**Problem formulation** - Description and significance of the problem/phenomenon studied; review of relevant theory and empirical work; problem statement	Page 4 Lines 2-23
**Purpose or research question** - Purpose of the study and specific objectives or questions	Page 4 Lines 24-30

Methods

**Qualitative approach and research paradigm** - Qualitative approach (e.g., ethnography, grounded theory, case study, phenomenology, narrative research) and guiding theory if appropriate; identifying the research paradigm (e.g., postpositivist, constructivist/ interpretivist) is also recommended; rationale**	Page 5 Lines 3-9
**Researcher characteristics and reflexivity** - Researchers' characteristics that may influence the research, including personal attributes, qualifications/experience, relationship with participants, assumptions, and/or presuppositions; potential or actual interaction between researchers' characteristics and the research questions, approach, methods, results, and/or transferability	Page 5 Lines 11-16
**Context** - Setting/site and salient contextual factors; rationale**	Page 5 Lines 19-23
**Sampling strategy** - How and why research participants, documents, or events were selected; criteria for deciding when no further sampling was necessary (e.g., sampling saturation); rationale**	Page 5 Line 25 - Page 6 Line 15
**Ethical issues pertaining to human subjects** - Documentation of approval by an appropriate ethics review board and participant consent, or explanation for lack thereof; other confidentiality and data security issues	Page 8 Line 27 - Page 9 Line 2
**Data collection methods** - Types of data collected; details of data collection procedures including (as appropriate) start and stop dates of data collection and analysis, iterative process, triangulation of sources/methods, and modification of procedures in response to evolving study findings; rationale**	Page 6 Lines 17-23

For peer review only - http://bmjopen.bmj.com/site/about/guidelines.xhtml

For peer review only

**Data collection instruments and technologies** - Description of instruments (e.g., interview guides, questionnaires) and devices (e.g., audio recorders) used for data collection; if/how the instrument(s) changed over the course of the study	Page 6 Lines 17-28
**Units of study** - Number and relevant characteristics of participants, documents, or events included in the study; level of participation (could be reported in results)	Page 6 Lines 17-23
**Data processing** - Methods for processing data prior to and during analysis, including transcription, data entry, data management and security, verification of data integrity, data coding, and anonymization/de-identification of excerpts	Page 6 Line 17 - Page 8 Line 12
**Data analysis** - Process by which inferences, themes, etc., were identified and developed, including the researchers involved in data analysis; usually references a specific paradigm or approach; rationale**	Page 8 Lines 14-20
**Techniques to enhance trustworthiness** - Techniques to enhance trustworthiness and credibility of data analysis (e.g., member checking, audit trail, triangulation); rationale**	Page 8 - 14-17

Results/findings

**Synthesis and interpretation** - Main findings (e.g., interpretations, inferences, and themes); might include development of a theory or model, or integration with prior research or theory	Page 9 Lines 4 -13
**Links to empirical data** - Evidence (e.g., quotes, field notes, text excerpts, photographs) to substantiate analytic findings	Page 9 Line 15 - Page 15 Line 13

Discussion

**Integration with prior work, implications, transferability, and contribution(s) to the field** - Short summary of main findings; explanation of how findings and conclusions connect to, support, elaborate on, or challenge conclusions of earlier scholarship; discussion of scope of application/generalizability; identification of unique contribution(s) to scholarship in a discipline or field	Page 15 Line 16 - Page 17 Line 7
**Limitations** - Trustworthiness and limitations of findings	Page 17 Lines 8-15

Other

**Conflicts of interest** - Potential sources of influence or perceived influence on study conduct and conclusions; how these were managed	Page 18 Line 12-14
**Funding** - Sources of funding and other support; role of funders in data collection, interpretation, and reporting	Page 18 Lines 19-20

*The authors created the SRQR by searching the literature to identify guidelines, reporting standards, and critical appraisal criteria for qualitative research; reviewing the reference lists of retrieved sources; and contacting experts to gain feedback. The SRQR aims to improve the transparency of all aspects of qualitative research by providing clear standards for reporting qualitative research.

For peer review only

For peer review only - http://bmjopen.bmj.com/site/about/guidelines.xhtml

**The rationale should briefly discuss the justification for choosing that theory, approach, method, or technique rather than other options available, the assumptions and limitations implicit in those choices, and how those choices influence study conclusions and transferability. As appropriate, the rationale for several items might be discussed together.

Reference:

O'Brien BC, Harris IB, Beckman TJ, Reed DA, Cook DA. **Standards for reporting qualitative research: a synthesis of recommendations.** *Academic Medicine*, Vol. 89, No. 9 / Sept 2014 DOI: 10.1097/ACM.0000000000000388

For peer review only
